# Supplementary material for: High-throughput tracking enables systematic phenotyping and drug repurposing in C. elegans disease models
Source: eLife. 2025 Jan 8;12:RP92491. doi: 10.7554/eLife.92491 (PMC11709427; doi:10.7554/eLife.92491)
Supplement: Supplementary file 2. [file elife-92491-supp2.pdf]

add-1(syb1598)

Strain name: PHX1598
Wild-type gene length: 1951bp
Mutation: 1539bp deletion at position 69 (second half of exon 1 to exon 5)

C. elegans Description

Function: Encodes cytoskeletal alpha-adducin; associated with cell membrane-mediated localised changes in cytoskeleton in response to activated signal transduction cascades

Expression: Coelomocytes; neurons; intestinal and rectal epithelia

Previously reported phenotypes: Defective short/long term memory and defective GLR-1 ionotropic glutamate receptor dynamics[1]

Human Orthologs:

ADD1; ADD3

Associated disease(s):
Artery disease; cerebral palsy, spastic quadriplegic, 3, autosomal recessive (AR) 617008; Hypertension, essential, salt-sensitive, mutational (Mu) 145500

Results

494/8289 significant features vs N2 (*p* < 0.05, block permutation t-test, 10000 permutations)

Key phenotype(s): increased curvature and angular velocity of the head

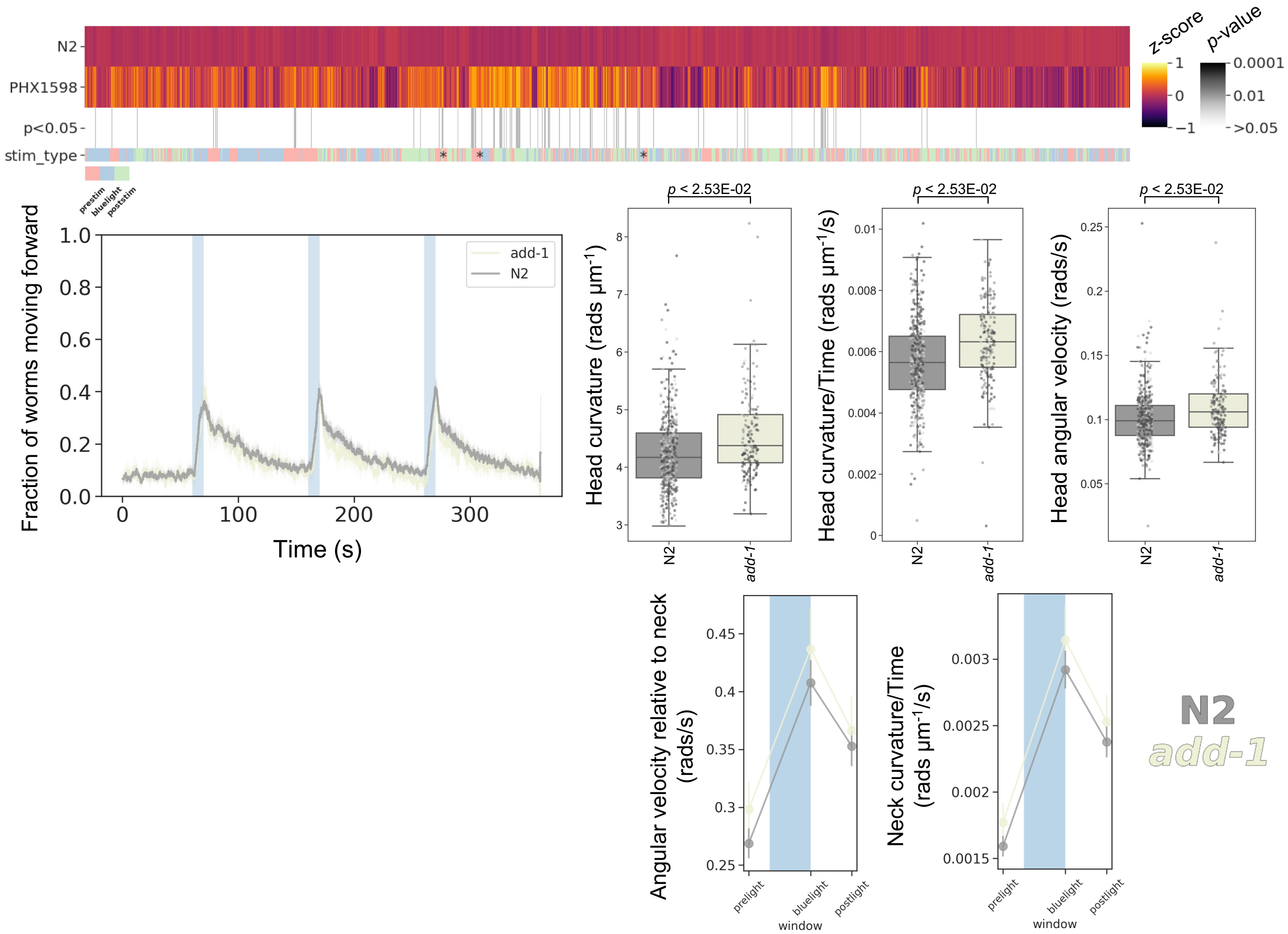

avr-14(syb1576)

Strain name: PHX1576
Wild-type gene length: 1111bp
Mutation: 628bp deletion at position 5 (exon 1 and start of exon 2)

C. elegans Description

Function: Localises to membrane and encodes glutamate-gated chloride channel activity (ivermectin sensitive)

Expression: Neurons and somatic nervous system

Previously reported phenotypes: Defective salt-chemotaxis behaviour and decreased locomotion on food[2-3]

Human Orthologs:

GLRA1, GLRB, GLRA3, GLRA4

Associated disease(s):
Hyperekplexia, hereditary 1, autosomal dominant (AD) or recessive (AR), 149400; Hyperekplexia 2, 614619 (AR)

Results

2717/8289 significant features vs N2 ($p < 0.05$, block permutation t-test, 10000 permutations)

Key phenotype(s): small; increased curvature of the head and midbody; decreased head foraging (radial velocity); attenuated blue light response (e.g., decreased increase in speed upon stimulation with blue light)

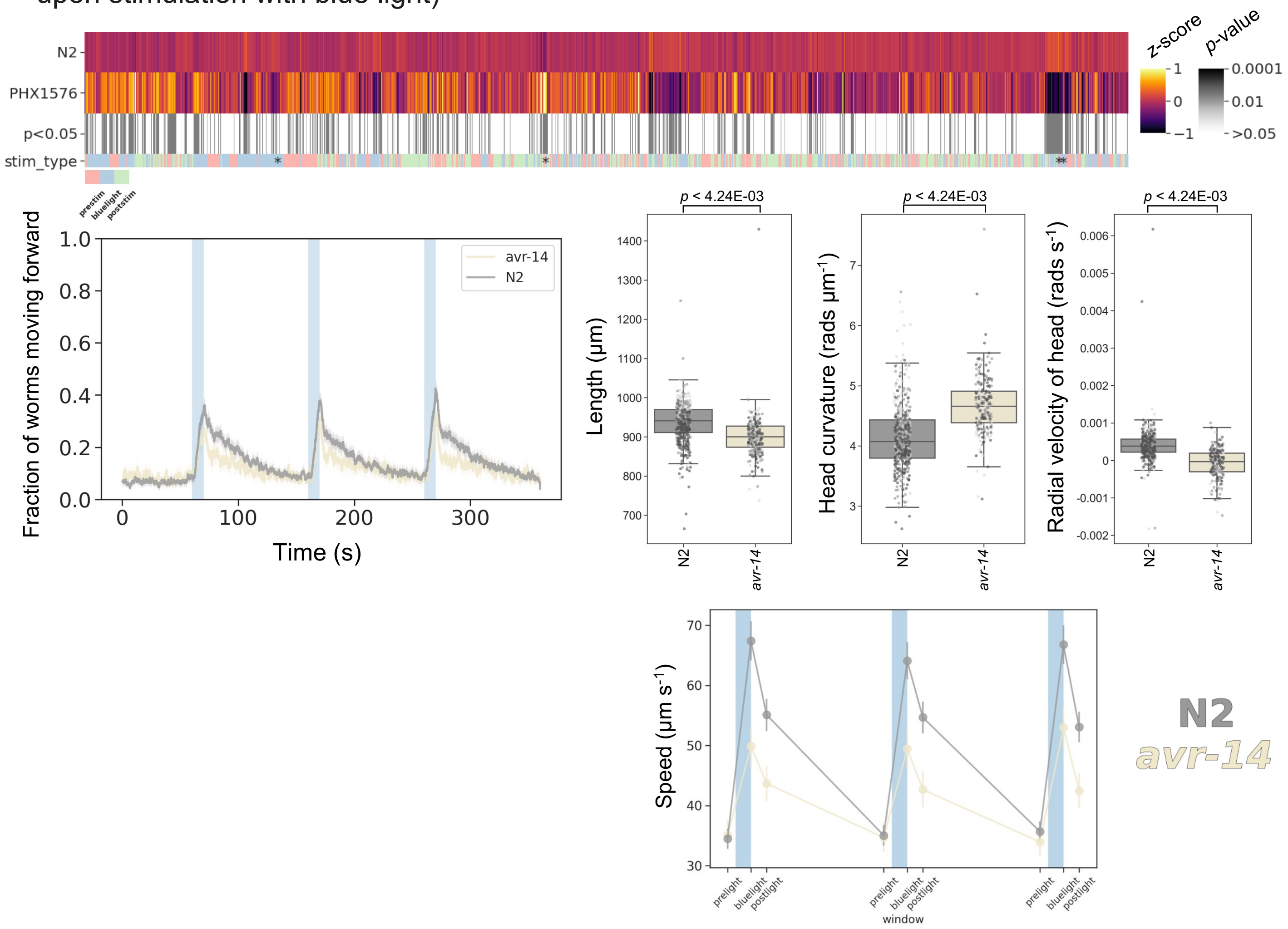

bbs-1(syb1588)

Strain name: PHX1588
Wild-type gene length: 1984bp
Mutation: 1412bp deletion at position 6 (exon 1 to start of exon 2)

C. elegans Description

Function: Involved in intraciliary retrograde transport, non-motile cilium assembly and protein localization to cilium; regulates fat storage, as such, *bbs-1* affects body size, feeding, chemosensation and is required for proper regulation of insulin secretion

Expression: Ciliated neurons and sensory neurons

Previously reported phenotypes: Increased secretion of insulin, biogenic amines and neuropeptides; abnormal dye filling; ivermectin resistance[4-5]

Human Orthologs:

BBS1

Associated disease(s):
Bardet-Biedl syndrome 1, autosomal recessive (AR) or digenic recessive (DR), 209900

Results

6124/8289 significant features vs N2 ($p < 0.05$, block permutation t-test, 10000 permutations)

Key phenotype(s): shorter and fatter; decreased curvature; deeper body bends (time derivative of curvature); hyperactive prior to simulation; delayed, but sustained, forward escape response and an increased, but short-lived, backward escape response to blue light

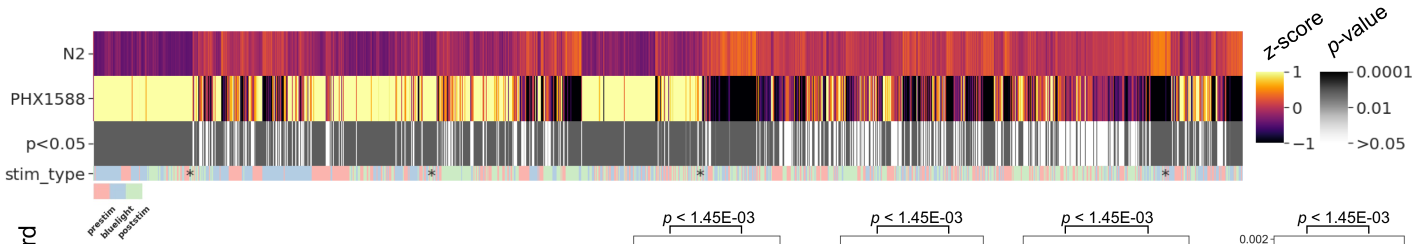

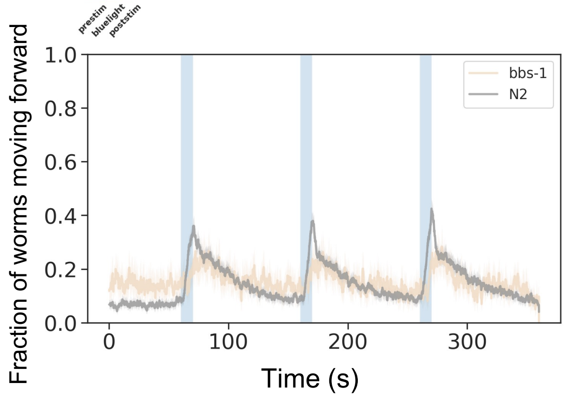

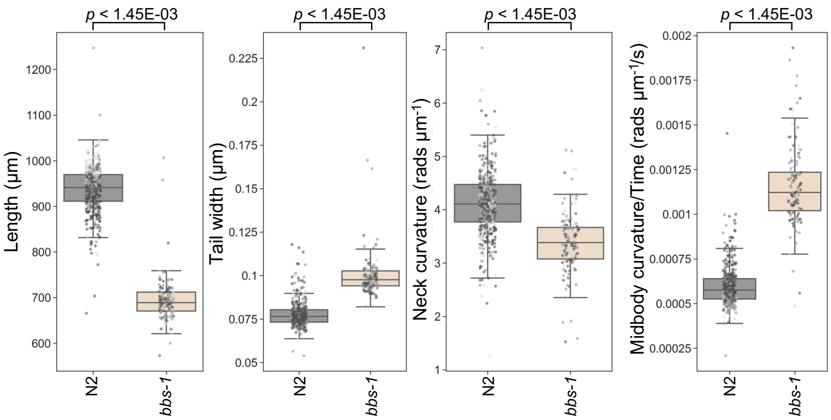

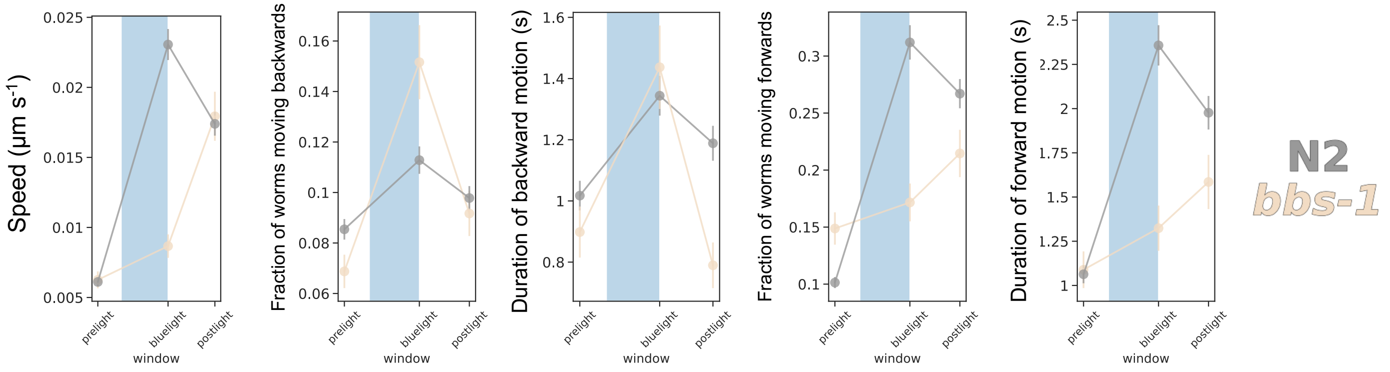

bbs-2(syb1547)

Strain name: PHX1547
Wild-type gene length: 2330bp
Mutation: 1513bp deletion at position 91 (second half exon 1 to half of exon 4)

C. elegans Description

Function: Involved in non-motile cillum assembly

Expression: Ciliated neurons and sensory neurons

Previously reported phenotypes: Body size, developmental delay and roaming defects[4]

Human Orthologs:

BBS2

Associated disease(s):
Bardet-Biedl syndrome 2, 615981 (AR); Retinitis pigmentosa 74, 616562 (AR)

Results

5693/8289 significant features vs N2 ($p < 0.05$, block permutation t-test, 10000 permutations)

Key phenotype(s): shorter and fatter; decreased curvature; deeper body bends (time derivative of curvature); hyperactive prior to stimulation; increased (but delayed) increase in speed, short lived backward escape response, and delayed (but sustained) forward escape response to blue light stimulation

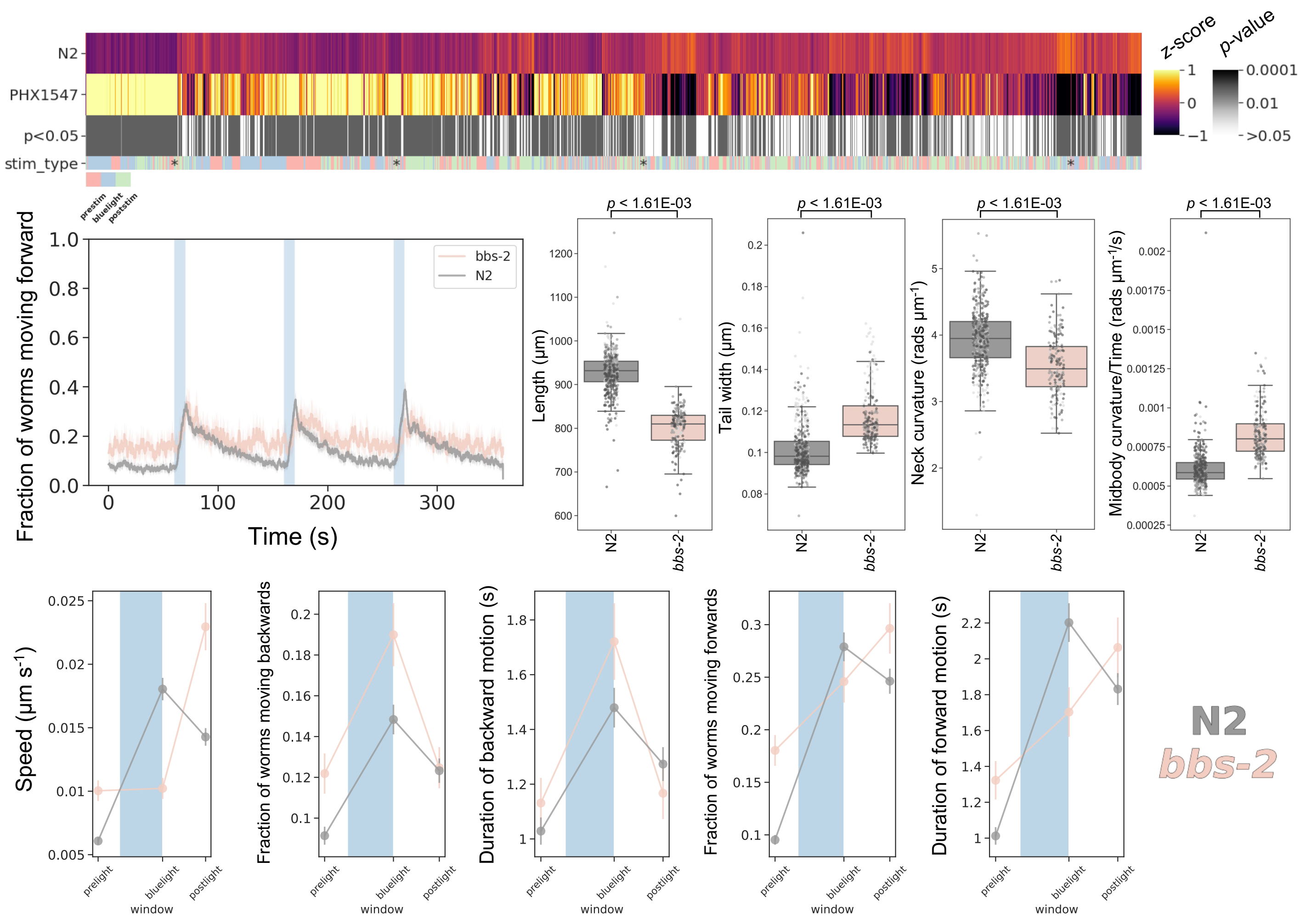

cat-2(syb1542)

Strain name: PHX1542
Wild-type gene length: 1683bp
Mutation: 1520bp deletion at position 33 (half of exon 1 to half of exon 3)

C. elegans Description

Function: Tyrosine hydroxylase activity (catecholamine synthesis)

Expression: Dopaminergic neurons and male-specific anatomy (spiricules and ray neurons)

Previously reported phenotypes: Decreased dopamine levels; defective food sensing; increased peak acceleration; increased variation in speed; increased habituation in response to nonlocalized mechanical stimulation[6-7]

Human Orthologs:

TH

Associated disease(s):
L-Dopa-responsive dystonia/Segawa syndrome, 605407 (AR); Obesity; Parkinsonism

Results

4479/8289 significant features vs N2 (p < 0.05, block permutation t-test, 10000 permutations)

Key phenotype(s): increased speed/curvature of head tip and decreased head foraging (radial velocity of head) while moving forward; hypersensitive to blue light stimulation (e.g., greater increase in speed and angular velocity); short backward escape response to blue light

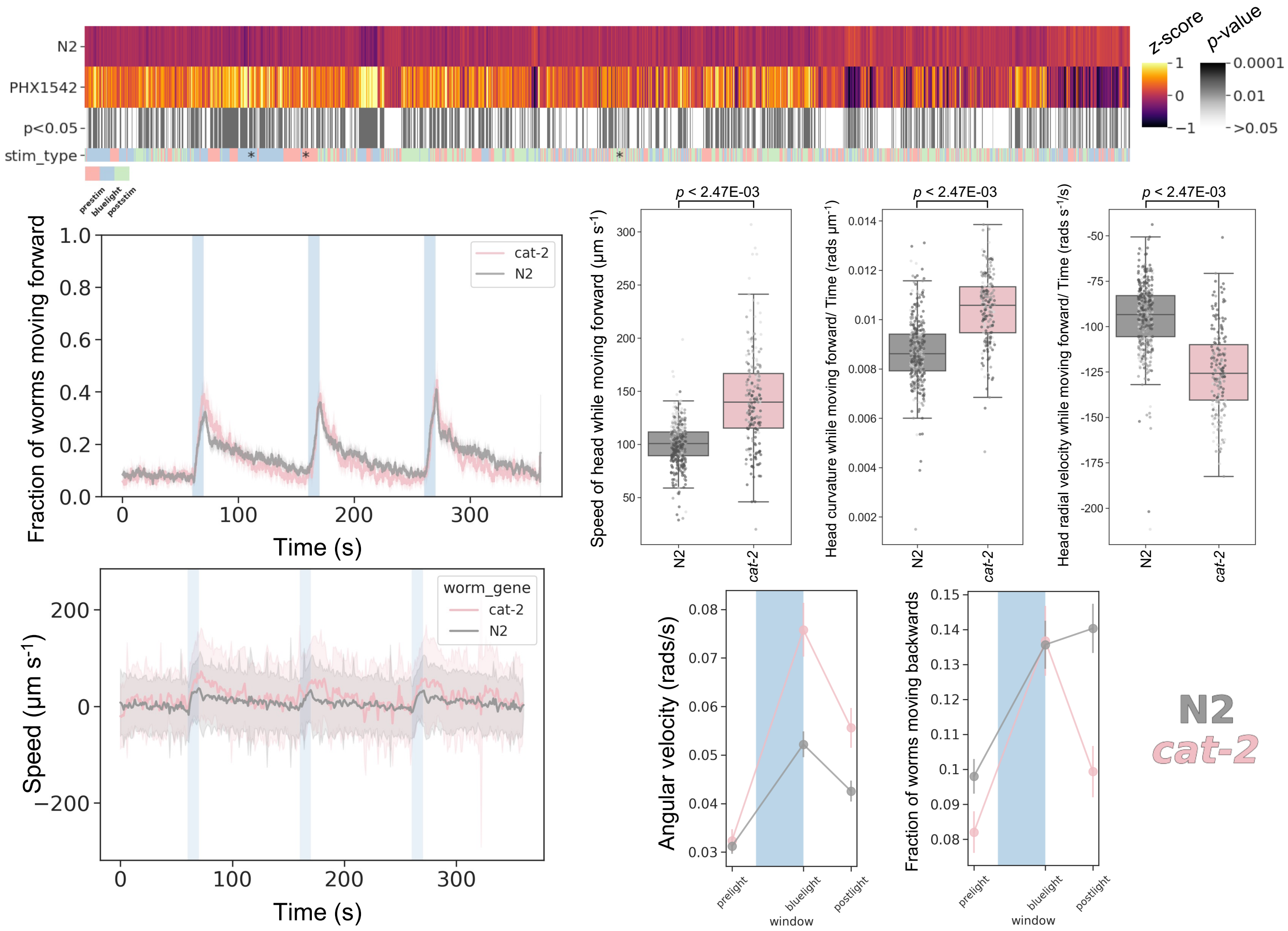

cat-4(syb1591)

Strain name: PHX1591
Wild-type gene length: 1647bp
Mutation: 891bp deletion at position 384 (exons 2-3 and half of exon 4)

C. elegans Description

Function: Putitative GTP cyclohydrolase I activity

Expression: Muscle cells; pharynx motor neurons; intestinal cells; dopaminergic, serotinergic, and mechanosensory neurons

Previously reported phenotypes: No previous phenotype reported

Human Orthologs:

GHC1

Associated diseases (from OMIM):
L-Dopa-responsive dystonia, with or without hyperphenylalaninemia, 128230 (AD, AR); Hyperphenylalaninemia, BH4 deficient, B, 233910 (AR); Bipolar disorder

Results

4182/8289 significant features vs N2 ($p < 0.05$, block permutation t-test, 10000 permutations)

Key phenotype(s): Developmental delay (2 days); small brood size; smaller; increased curvature; decreased speed; less active; strong photophobic escape response; short duration of backwards escape response

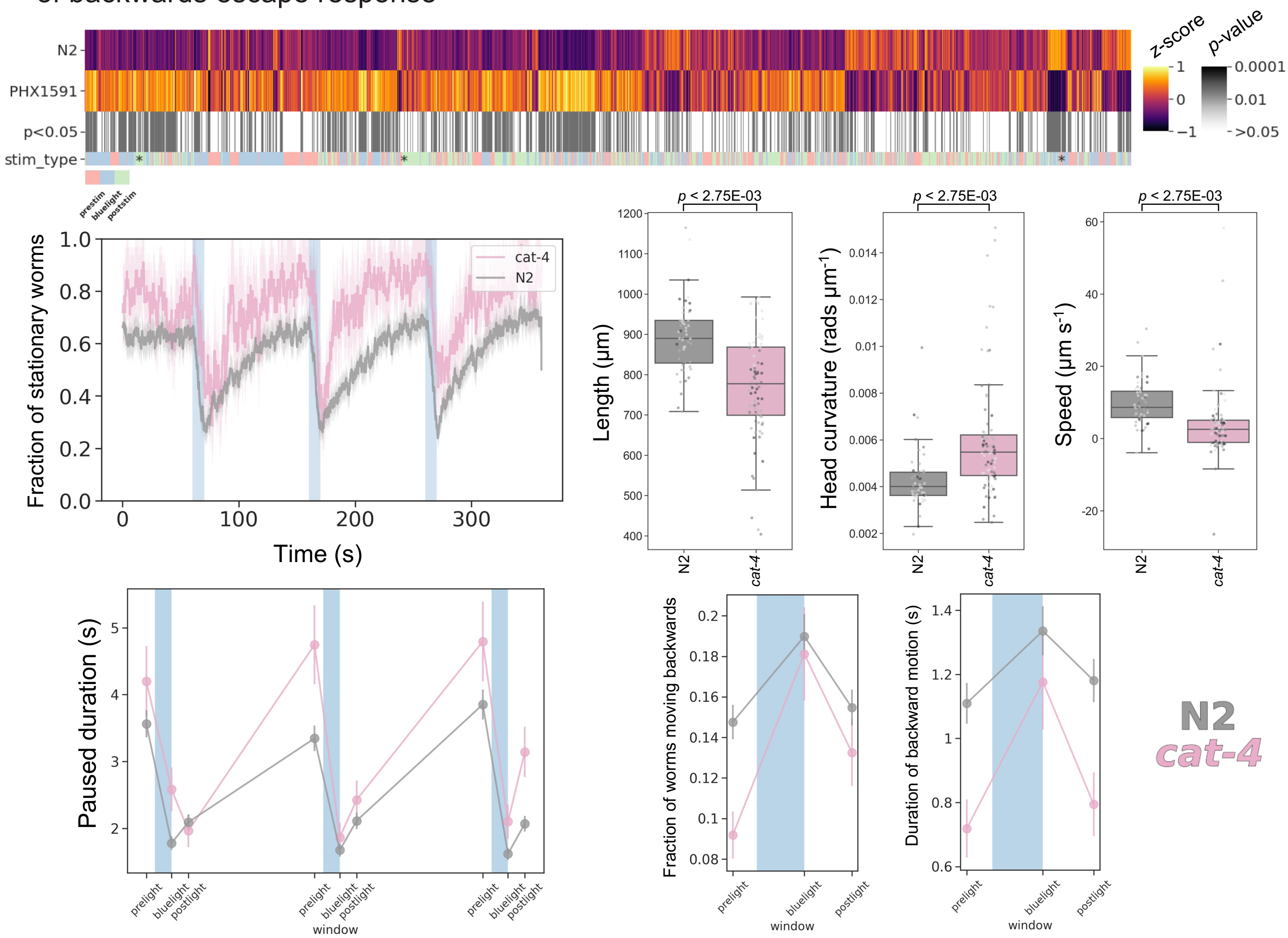

dys-1(syb1688)

Strain name: PHX1688
Wild-type gene length: 30990bp
Mutation: 30716bp deletion at position 59 (all of the F32B4.10 transcript)

C. elegans Description

Function: Localizes to dystrobrevin complex

Expression: Body wall musculature; head muscle; pharyngeal muscle; vulval muscle

Previously reported phenotypes: Subtle hyperactive locomotion; exaggerated head bending; slight hypercontractions; abnormal burrowing[8-9]

Human Orthologs:

DMD

Associated disease(s):
Duchenne muscular dystrophy, X-linked recessive (XLR) 310200; Becker muscular dystrophy, 300376 (XLR); Cardiomyopathy dilated, 302045 (XLR)

Results

1038/8289 significant features vs N2 ($p < 0.05$, block permutation t-test, 10000 permutations)

Key phenotype(s): Developmental delay (4 days); increased curvature, angular velocity and speed of head; increased sensitivity to blue light stimuli (e.g., greater increase in speed/forward motion upon stimulation); strongly attenuated backward escape response to blue light stimulation

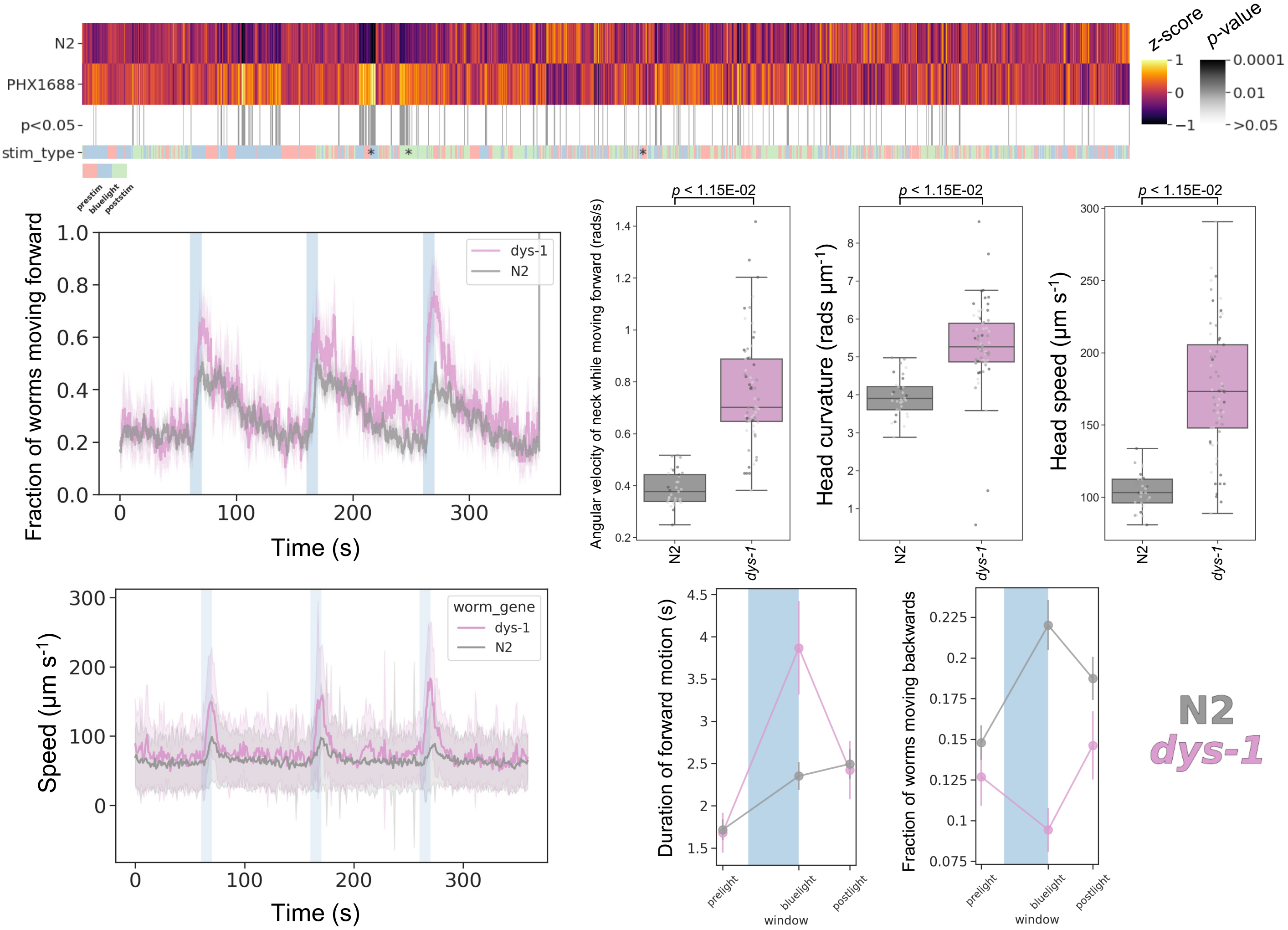

figo-1(syb1562)

Strain name: PHX1562
Wild-type gene length: 1633bp
Mutation: 1038bp deletion at position 18 (removing the first 3 exons)

C. elegans Description

Function: Putative phosphoric ester hydrolase activity

Expression: GABAergic neurons; body wall musculature; head neurons; intestine and tail neurons

Previously reported phenotypes: No previous phenotype reported

Human Orthologs:

FIG4

Associated disease(s):

Amyotrophic lateral sclerosis 11, 612577 (AD); Charcot-Marie-Tooth disease, type 4J, 611228 (AR); Polymicrogyria, bilateral temporooccipital, 612691 (AR); Yunis-Varon syndrome, 216340 (AR)

Results

2799/8289 significant features vs N2 ($p < 0.05$, block permutation t-test, 10000 permutations)

Key phenotype(s): Developmental delay (2 days); increased angular velocity/curvature of the head, neck and tail; deeper body bends (time derivative of curvature); sustained forward, but attenuated backward, escape response to blue light stimulation; greater increase in speed upon repeated blue light stimulation

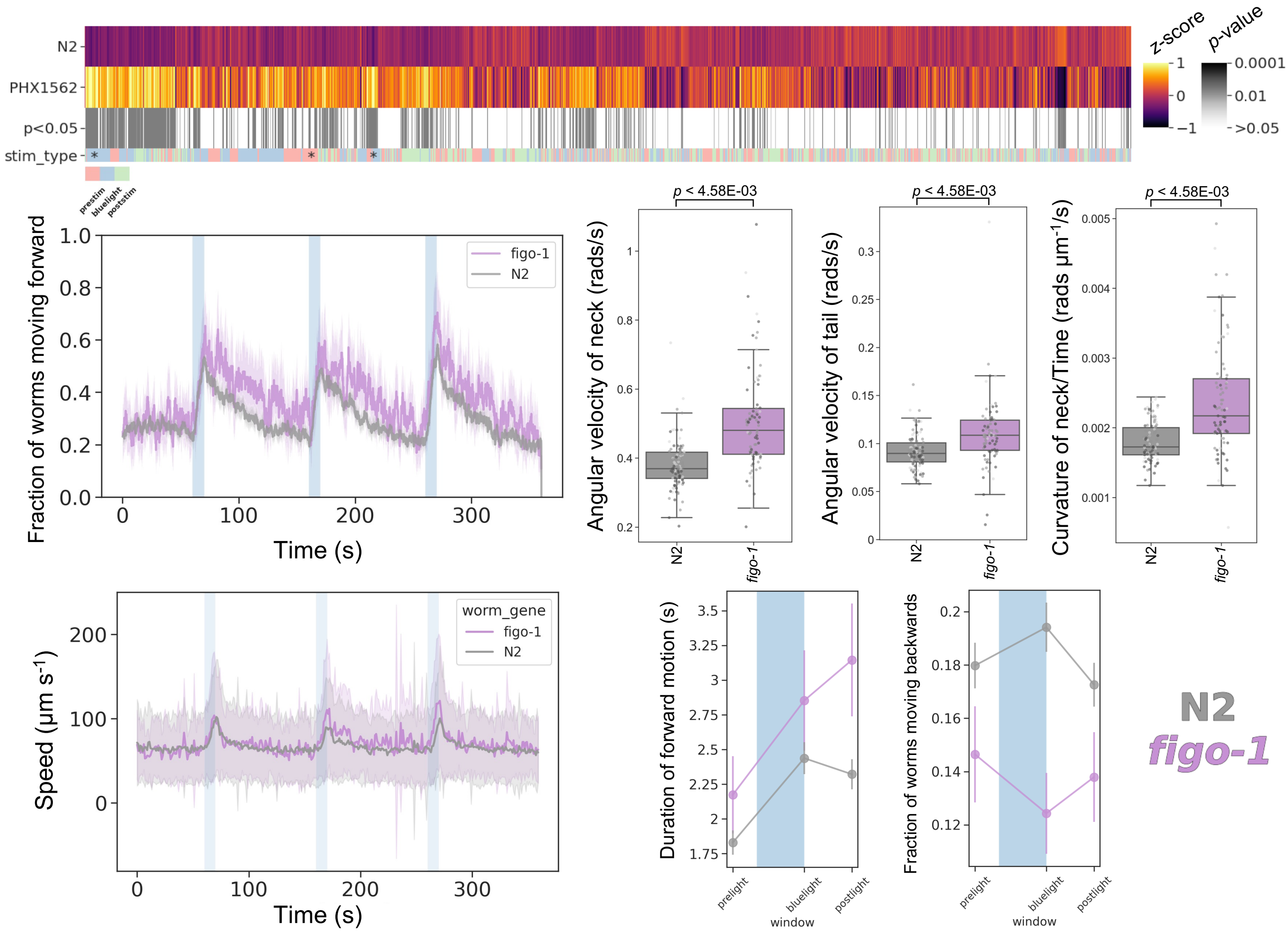

glc-2(syb1557)

Strain name: PHX1557
Wild-type gene length: 1512bp
Mutation: 1269bp deletion at position 106 (first 4 exons)

C. elegans Description

Function: Glutamate-gated Chloride channel (GLC-2), capable of forming homomeric glutamate-activated channels and heteromeric glutamate-activated channels with GLC-1; avermectins or anthelmintics inhibit pharyngeal pumping

Expression: Pharyngeal pm4 muscle

Previously reported phenotypes: No previous phenotype reported

Human Orthologs:

GLRA1; GLRB

Associated disease(s):
Hyperekplexia, hereditary 1, 149400 (AD or AR); Hyperekplexia 2, 614619 (AR)

Results

2413/8289 significant features vs N2 (*p* < 0.05, block permutation t-test, 10000 permutations)

Key phenotype(s): Smaller; increased curvature and speed of the head; hyperactive; increased fraction and duration of forward motion; attenuated backward escape response; diminished blue light response (e.g., change in angular velocity upon stimulation is decreased)

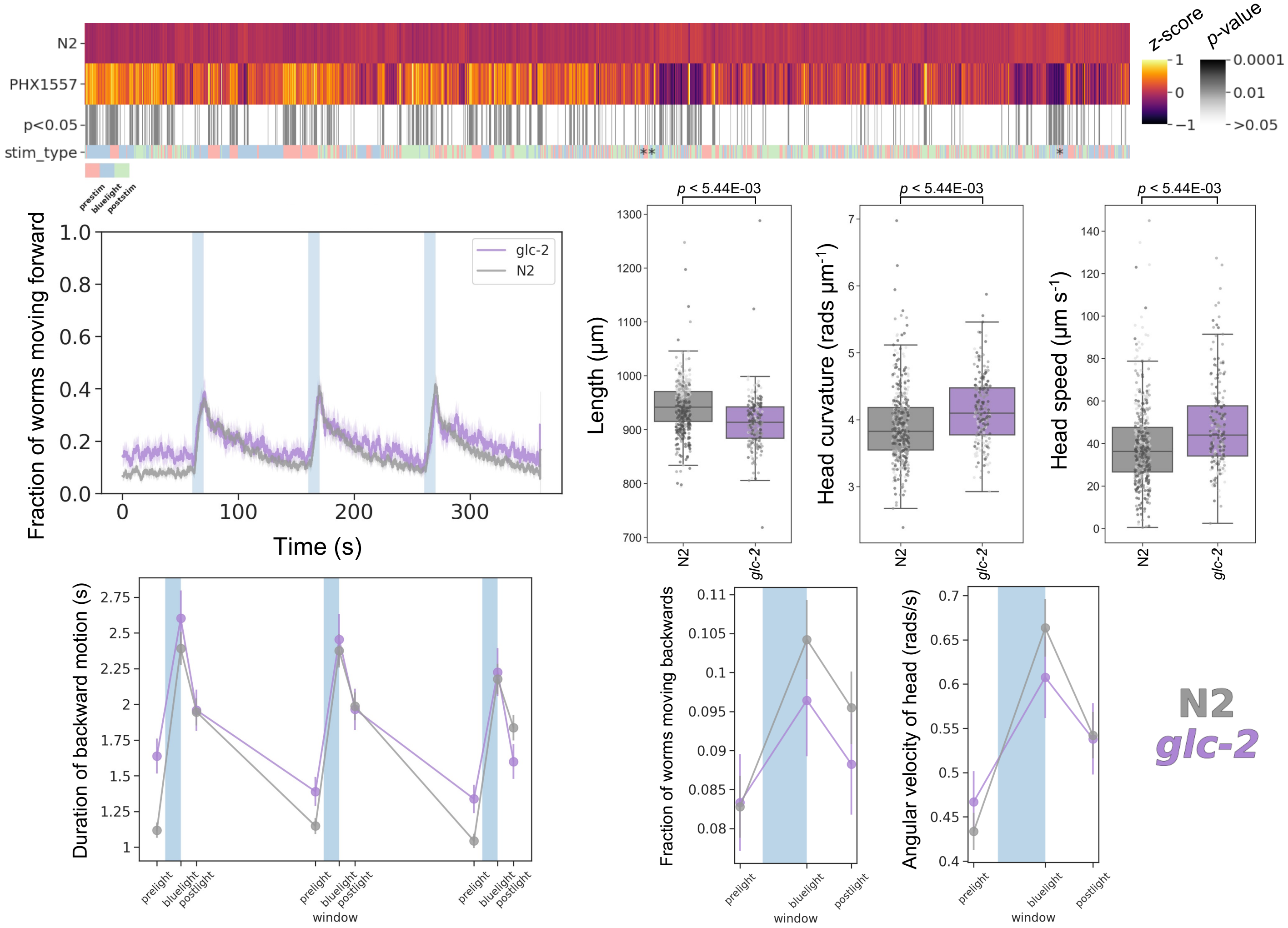

glr-1(syb1556)

Strain name: PHX1556
Wild-type gene length: 1488bp
Mutation: 979bp deletion at position 10 (exon 1 to beginning of exon 4)

C. elegans Description

Function: Localizes to several cellular components, including: ionotropic glutamate receptor complex; neuron projection membrane and perinuclear endoplasmic reticulum

Expression: Ganglia; neurons and ventral nerve cord

Previously reported phenotypes: Gain of function mutants are resistant to dopamine-induced food paralysis and have higher reversal rate; loss of function mutants are defective in light nose touch response; resistance to fluoxetine-induced paralysis[10-12]

Human Orthologs:

GRIA3, GRIK2

Associated disease(s):
Intellectual disability, 94, 300699 (XLR); Intellectual disability, 6, 611092 (AR); Huntington's

Results

3820/8289 significant features vs N2 ($p < 0.05$, block permutation t-test, 10000 permutations)

Key phenotype(s): Longer; increased foraging; increased curvature/angular velocity of all body segments; increased speed; hyperactive; attenuated backward escape response to blue light

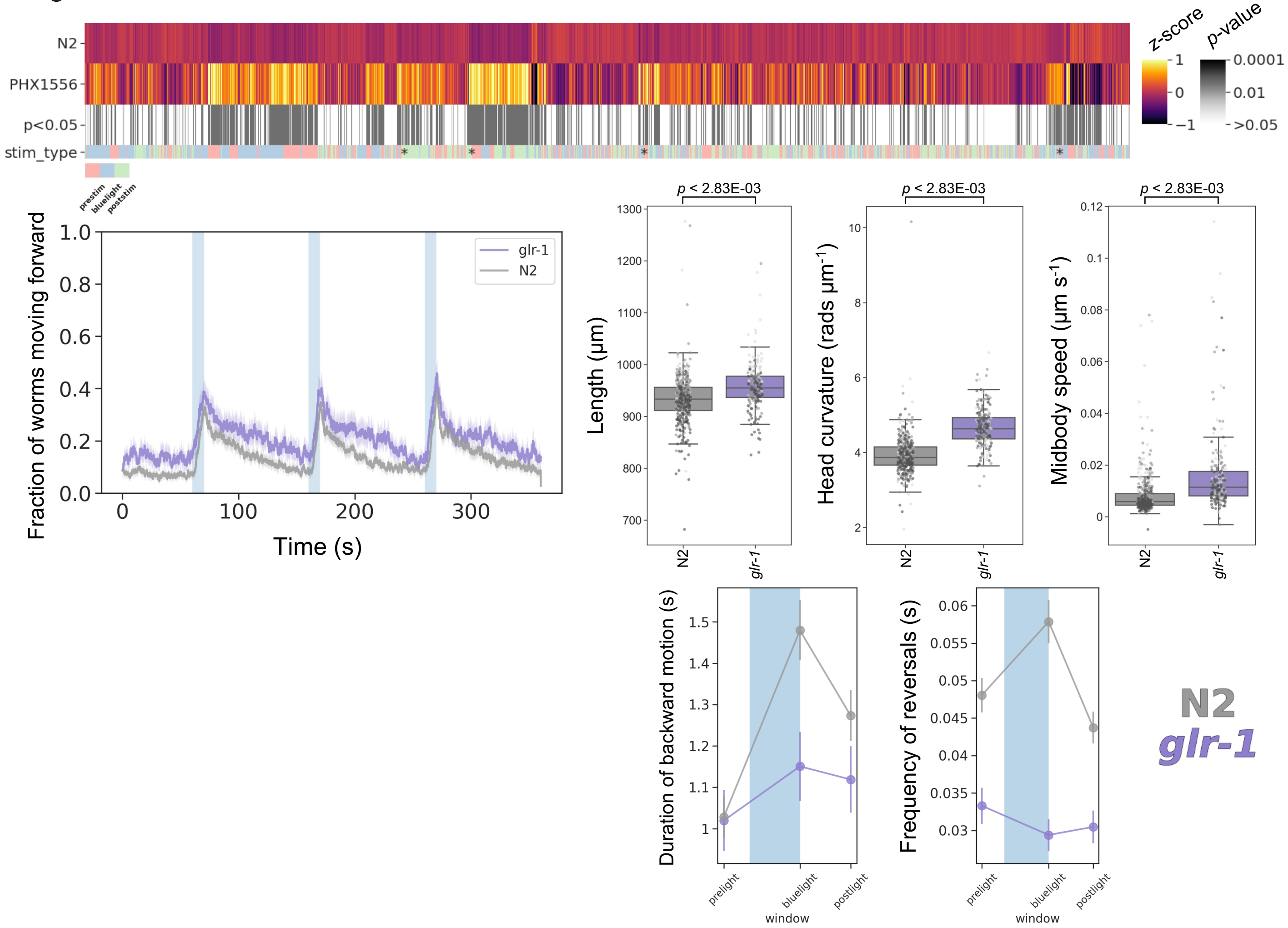

glr-4(syb1518)

Strain name: PHX1518
Wild-type gene length: 2124bp
Mutation: 1356bp deletion at position 103 (end of exon 1 to exon 8)

C. elegans Description

Function: NMDA-receptor subunit (encodes a putative non-NMDA ionotropic glutamate receptor subunit)

Expression: DA9; SABVL; SABVR and VB neurons

Previously reported phenotypes: Aldicarb resistance[13]

Human Orthologs:

GRIK2

Associated disease(s):
Intellectual disability, 6, 611092 (AR)

Results

0/8289 significant features vs N2 ($p < 0.05$, block permutation t-test, 10000 permutations)

Key phenotype(s): No significant phenotype detected

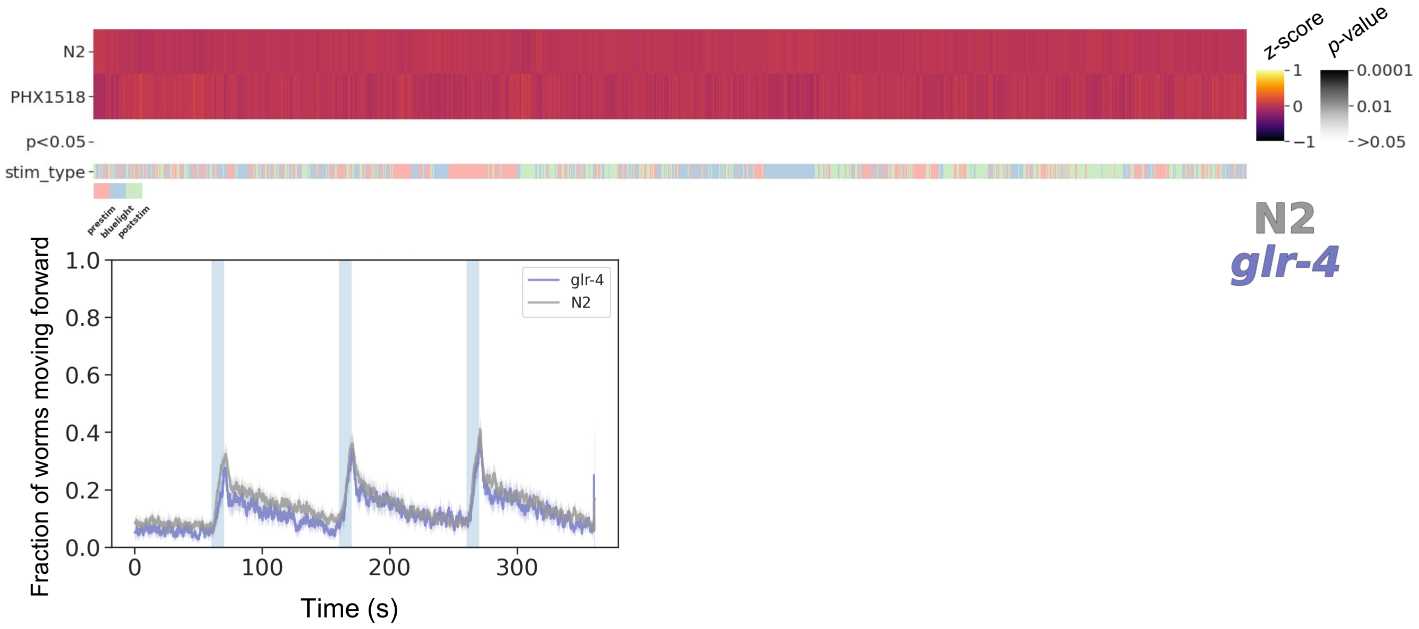

gpb-2(syb1577)

Strain name: PHX1577
Wild-type gene length: 3009bp
Mutation: 2408bp deletion at position 140 (all exons)

C. elegans Description

Function: G protein-coupled acetylcholine receptor signalling pathway; egg-laying

Expression: Body wall musculature; nerve ring; pharyngeal muscle cell and vulval muscle

Previously reported phenotypes: Slippery corpus; slightly longer; increased sinusoidal and deeper body bends; defects in egg laying and pharyngeal pumping; arecoline sensitive[14-16]

Human Orthologs:

GNB5

Associated disease(s):
Intellectual developmental disorder with cardiac arrhythmia, 617173 (AR); Language delay and ADHD/cognitive impairment with or without cardiac arrhythmia, 617182 (AR)

Results

2954/8289 significant features vs N2 ($p < 0.05$, block permutation t-test, 10000 permutations)

Key phenotype(s): Developmental delay (2 days); increased curvature/angular velocity of hips and tail; increased sinusoidal movements (time derivative of curvature); hyperactive; stronger, more sustained, backward escape response; hypersensitive to blue light

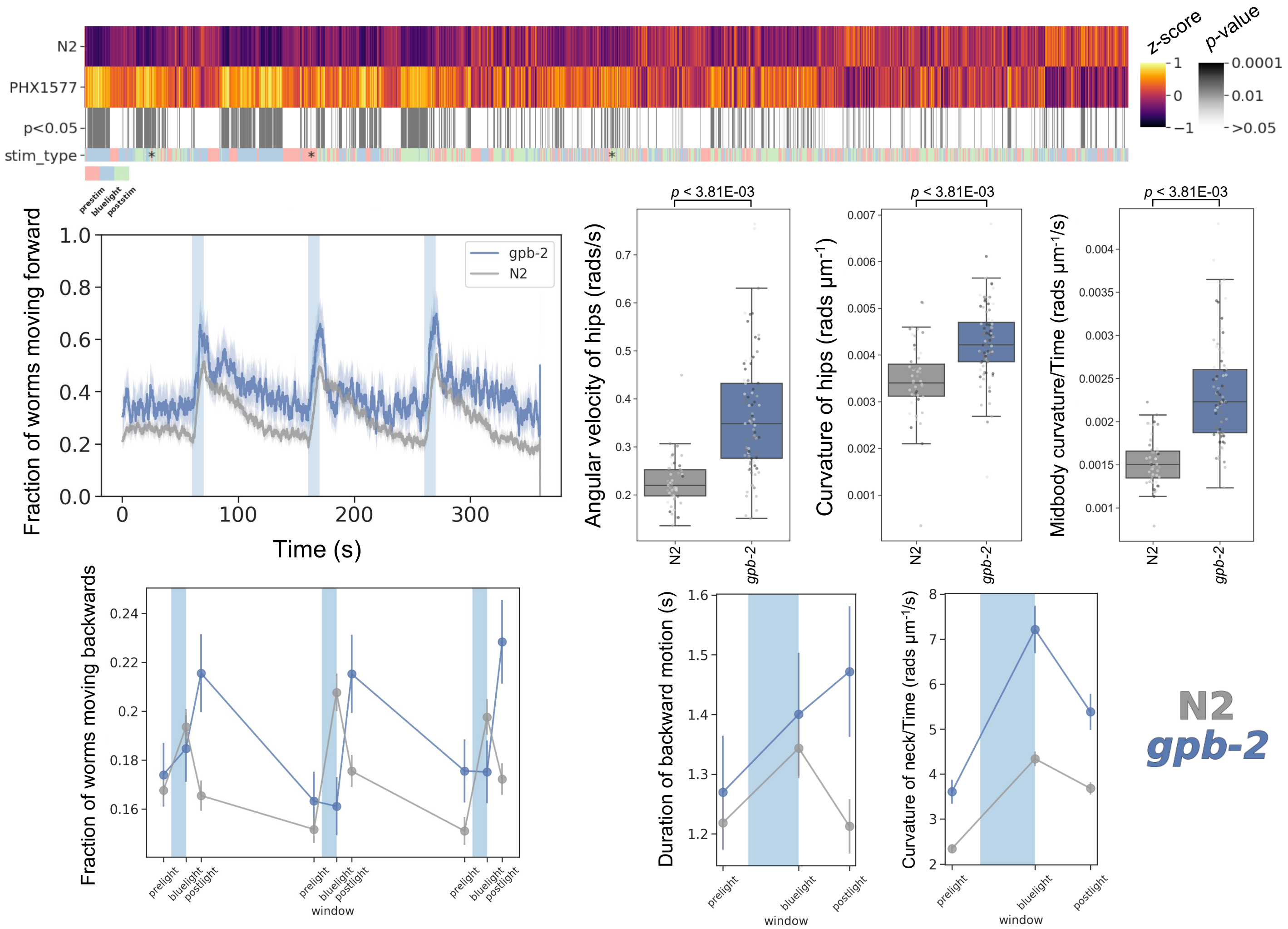

kcc-2(syb2673)

Strain name: PHX2673
Wild-type gene length: 4387bp
Mutation: 3919bp deletion at position 45 (first 3 exons and most piwi-interacting RNAs)

C. elegans Description

Function: Potassium-chloride c-transporter (partially redundant with abts-1); involved in chloride/potassium ion transport and regulation of GABAergic synaptic transmission

Expression: Body wall musculature; egg-laying apparatus; hermaphrodite distal tip cell; intestine and neurons

Previously reported phenotypes: Decreased egg laying; shorter; serotonin resistant; displays an excitatory, not inhibitory, response to the GABAa agonist muscimol[17-18]

Human Orthologs:

SLC12A5, SLC12A6

Associated disease(s):

Epilepsy, idiopathic generalized, 14, 616685 (AD); Epileptic encephalopathy, early infantile, 34, 616645 (AR); Agenesis of the corpus callosum with peripheral neuropathy, 218000 (AR)

Results

3246/8289 significant features vs N2 ($p < 0.05$, block permutation t-test, 10000 permutations)

Key phenotype(s): Developmental delay (2 days); increased curvature and speed of head; decreased sinusoidal undulations (time derivative of midbody curvature); decreased angular velocity; hypoactive; sustained forward escape response; attenuated blue light response

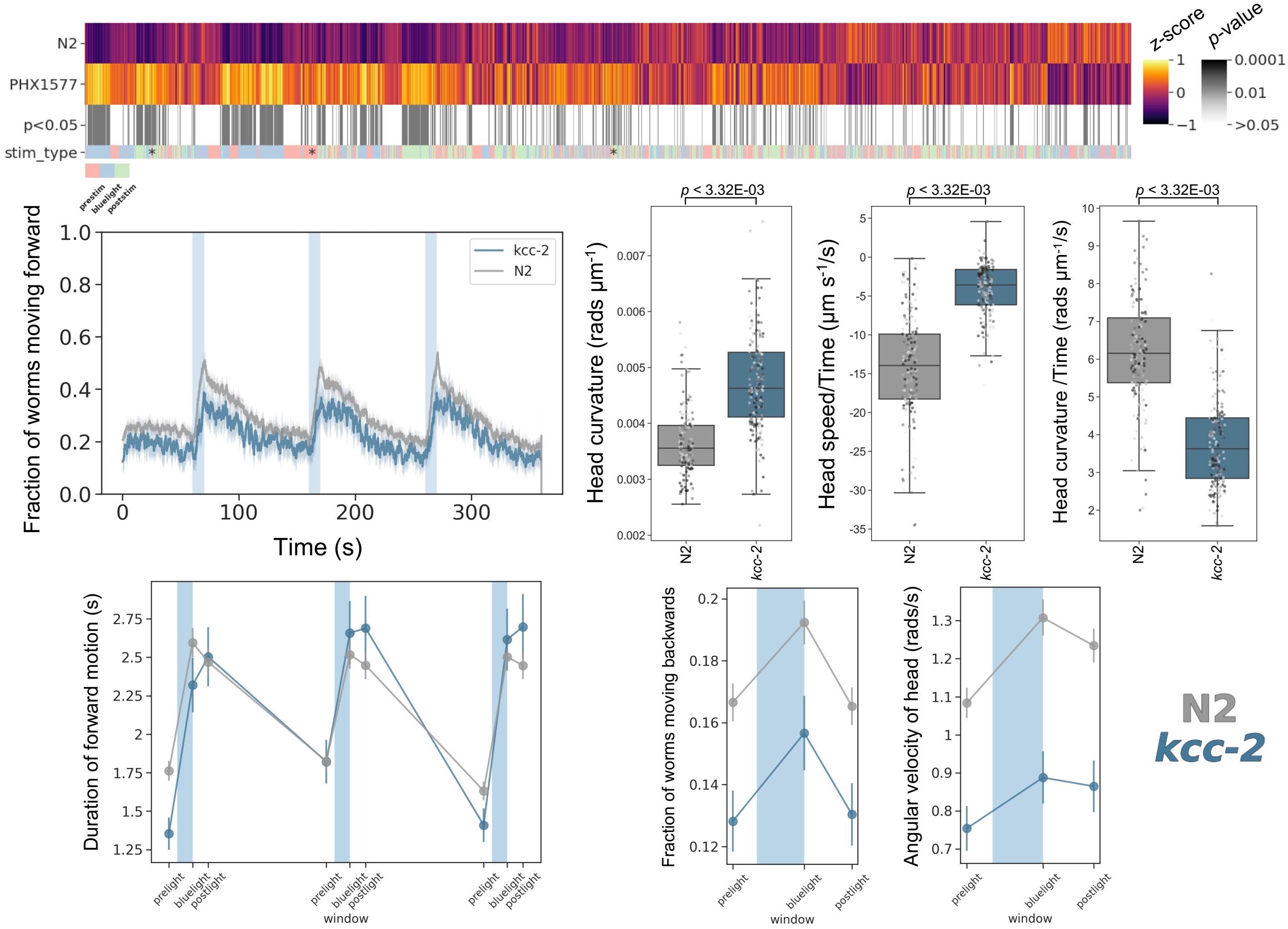

mpz-1(syb1522)

Strain name: PHX1522
Wild-type gene length: 36850bp
Mutation: 35787bp deletion at position 423 (entire gene)

C. elegans Description

Function: Encodes multi-PDZ domain scaffold protein that exhibits GTPase activating protein binding activity

Expression: Body wall musculature; nerve ring; neurons and vulval muscle

Previously reported phenotypes: *mpz-1* RNAi reduces 5-HT stimulated egg laying[19]

Human Orthologs:

MPDZ

Associated disease(s) :
Hydrocephalus, nonsyndromic, 2, 615219 (AR)

Results

4957/8289 significant features vs ($p < 0.05$, block permutation t-test, 10000 permutations)

Key phenotype(s): Shorter; decreased curvature of the head/neck but increased curvature of hips/midbody; increased speed (hypersensitive to blue light); hyperactive; sustained forward escape response; increased rate of reversals (fraction of backwards moving worms)

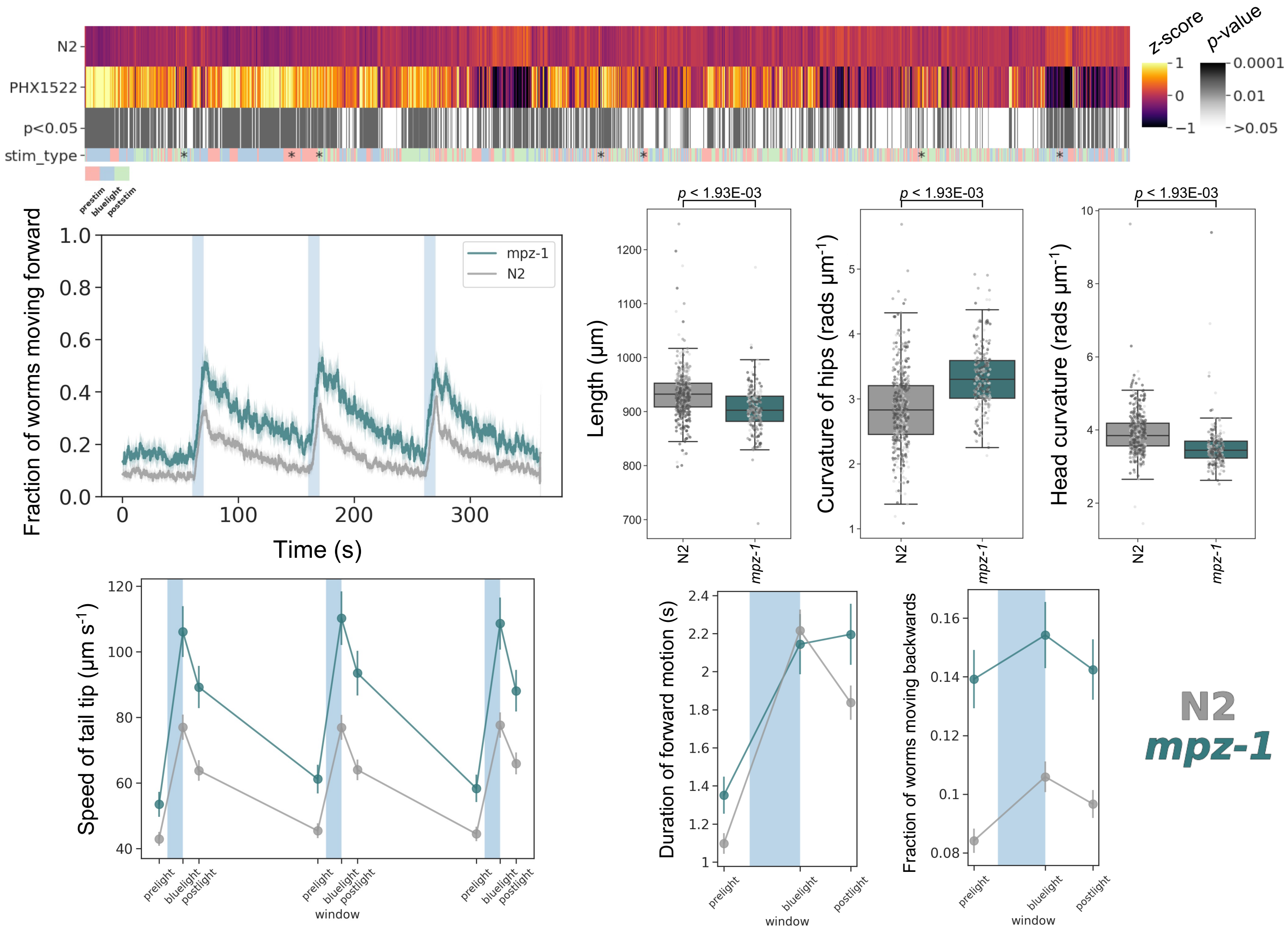

nca-2(syb1612)

Strain name: PHX1612
Wild-type gene length: 8377bp
Mutation: 6003bp deletion at position 20 (first 13.5 exons)

C. elegans Description

Function: Cation leak channel activity; interacts with *unc-77* and *unc-80*

Expression: Intestine; pharyngeal neurons and somatic nervous system

Previously reported phenotypes: Locomotion deficits; fainter in liquid; sensitive to halothane and isoflurane[15]

Human Orthologs:

NALCN

Associated disease(s):
Congenital contractures of the limbs and face, hypotonia, and developmental delay, 616266 (AD); Hypotonia, infantile, with psychomotor retardation and characteristic facies 1, 615419 (AR)

Results

2522/8289 significant features vs N2 ($p < 0.05$, block permutation t-test, 10000 permutations)

Key phenotype(s): Smaller; decreased speed; increased curvature/angular velocity of head only; no difference in baseline locomotion but an increased, short-lived, reversal response to blue light; increased post-stimulation pausing, “fainting” phenotype

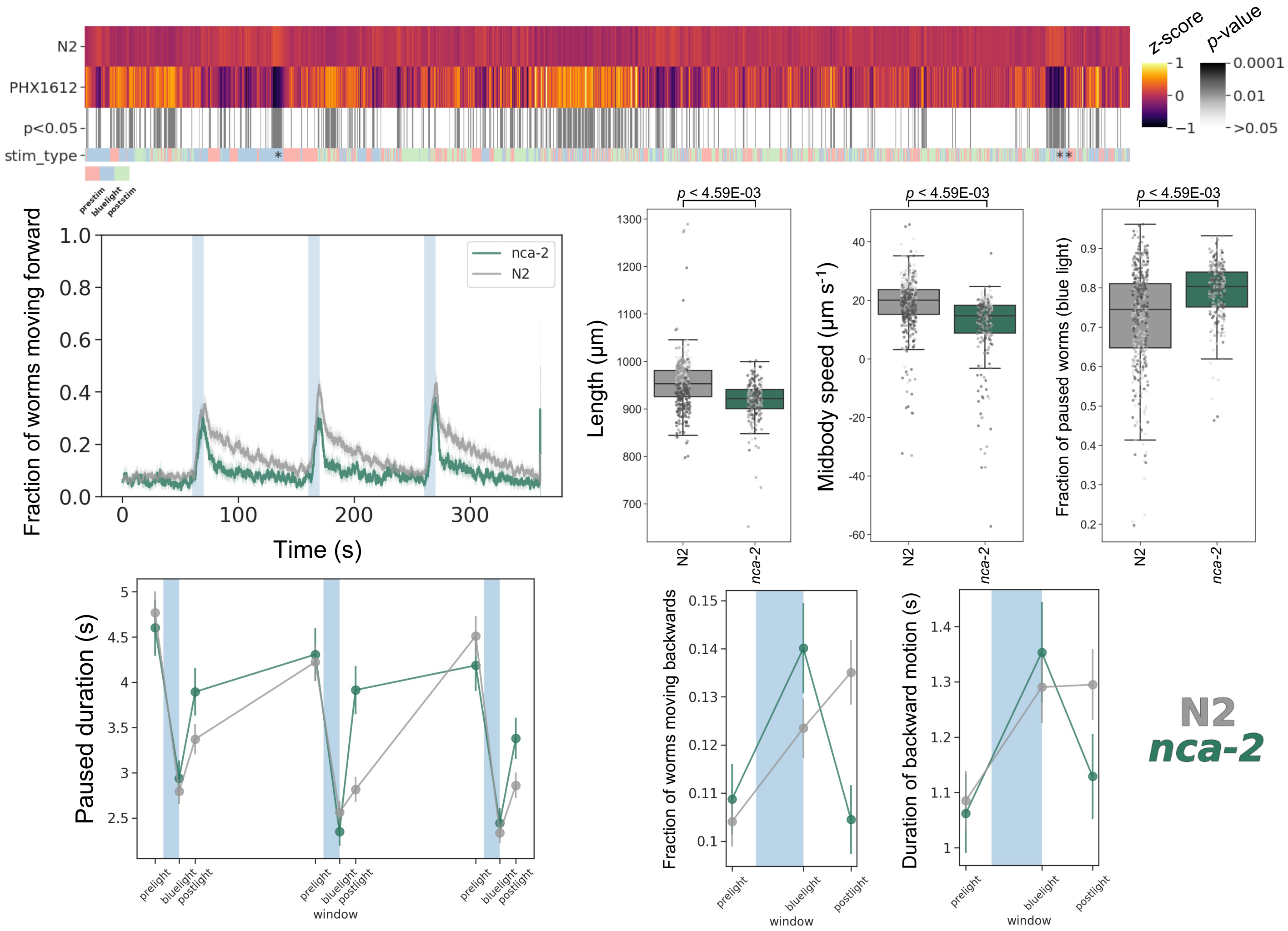

pink-1(syb1546)

Strain name: PHX1546
Wild-type gene length: 1926bp
Mutation: 1381bp deletion at position 157 (second half exon 2 to exon 6)

C. elegans Description

Function: Serine/threonine-specific protein kinase

Expression: Hermaphrodite distal tip cell; intestine; neurons; pharynx and vulva

Previously reported phenotypes: Required for: normal response to oxidative stress; mitochondrial homeostasis; correct CAN neurite outgrowth and wild type brood sizes. Mutant phenotypes are suppressed by mutation in *lrk-1* (LRRK2 ortholog)[20-22]

Human Orthologs:

PINK1

Associated disease(s):
Parkinson disease 6, early onset, 605909 (AR)

Results

0/8289 significant features vs N2 ($p < 0.05$, block permutation t-test, 10000 permutations)

Key phenotype(s): No significant phenotype detected

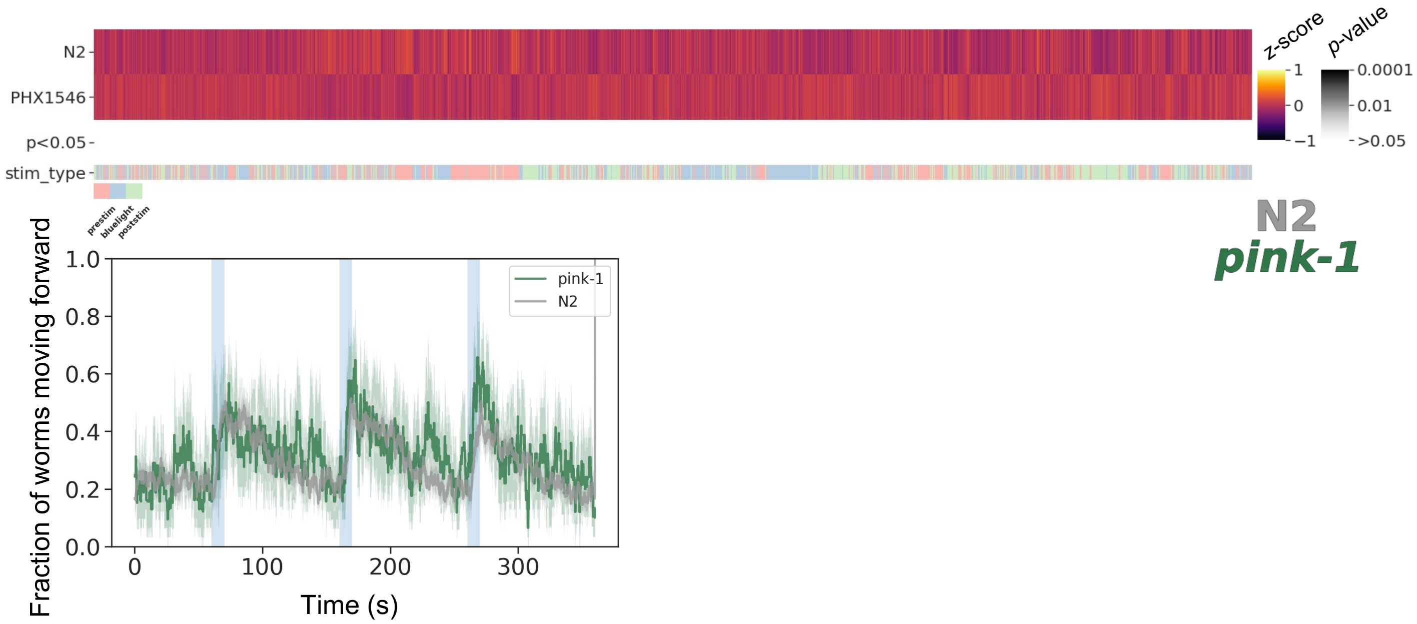

snf-11(syb2683)

Strain name: PHX2683
Wild-type gene length: 2283bp
Mutation: 1543bp deletion at position 16 (exon 1 to second half of exon 4)

C. elegans Description

Function: Sodium/chloride ion-coupled high affinity GABA transporter; involved in gamma-aminobutyric acid transport

Expression: GLR; enteric muscle; head mesodermal cell; neurons and ventral nerve cord

Previously reported phenotypes: Abnormal head foraging and sinusoidal movements[15]

Human Orthologs:

SLC6A1

Associated disease(s):
Myoclonic-atonic epilepsy, 616421 (AD)

Results

3601/8289 significant features vs N2 (*p* < 0.05, block permutation t-test, 10000 permutations)

Key phenotype(s): slightly less active; low frequency head bends (decreased time derivative of curvature); increased speed of head; increased foraging behaviour (time derivative of radial velocity); strong, but short-lived, backwards escape response to blue light stimulation

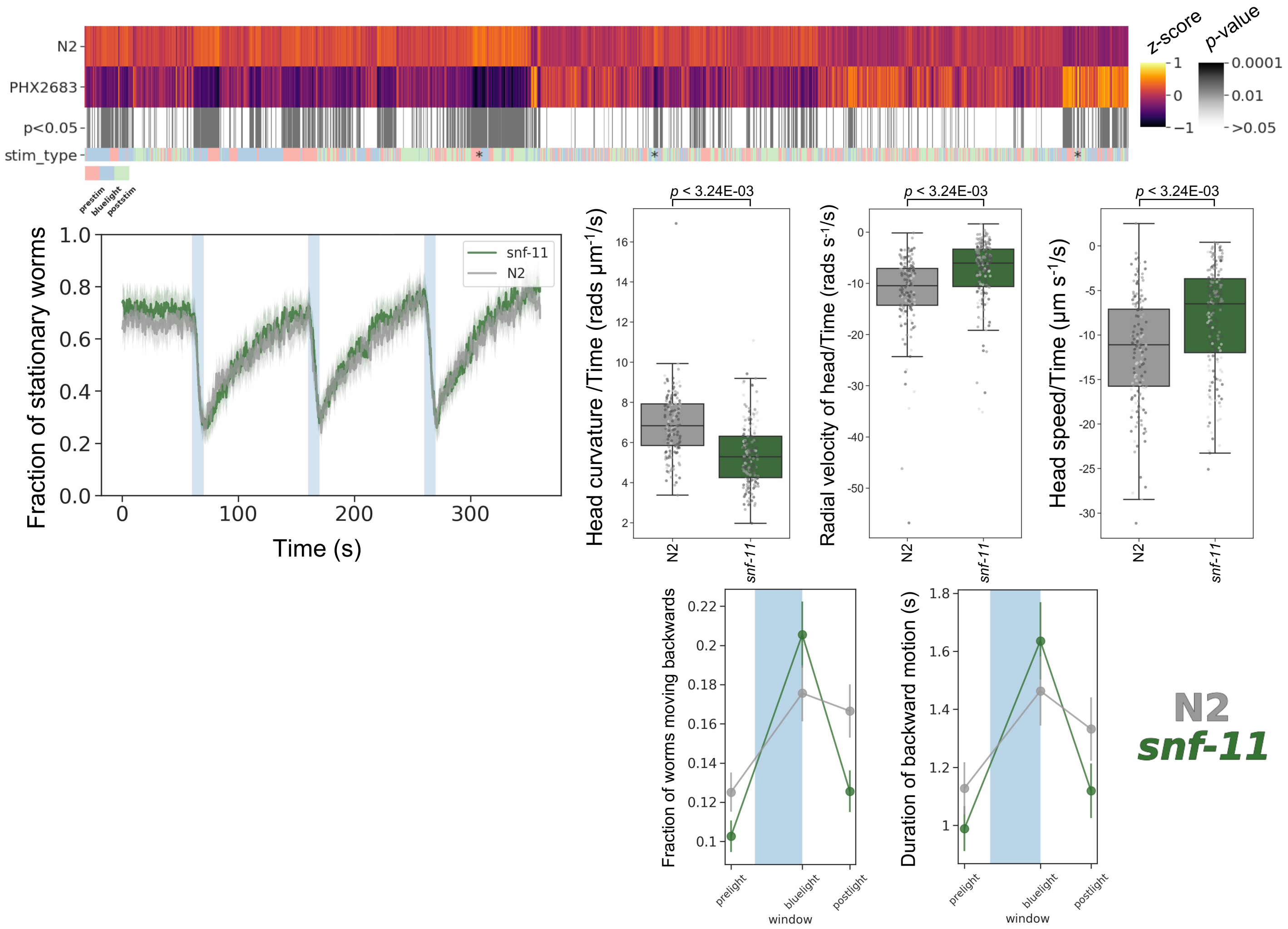

snn-1(syb2695)

Strain name: PHX2695
Wild-type gene length: 2682bp
Mutation: 1835bp deletion at position 22 (2 exons)

C. elegans Description

Function: Synapsin, involved in neuromuscular synaptic transmission

Expression: All neurons; localises to presynaptic regions

Previously reported phenotypes: Aldicarb resistance; defective synaptic vesicle clustering[13,23]

Human Orthologs:

SYN1;SYN2

Associated disease(s):

Epilepsy, X-linked recessive and dominant (XLR, XLD), with variable learning disabilities and behaviour disorders, 300491; Schizophrenia susceptibility, 181500 (AD)

Results

1633/8289 significant features vs N2 (*p* < 0.05, block permutation t-test, 10000 permutations)

Key phenotype(s): Less active; shallow head movements (decreased time derivative of curvature); increased head speed; increased foraging behaviour (time derivative of radial velocity); increased reversal response to blue light that diminishes upon repeated exposure

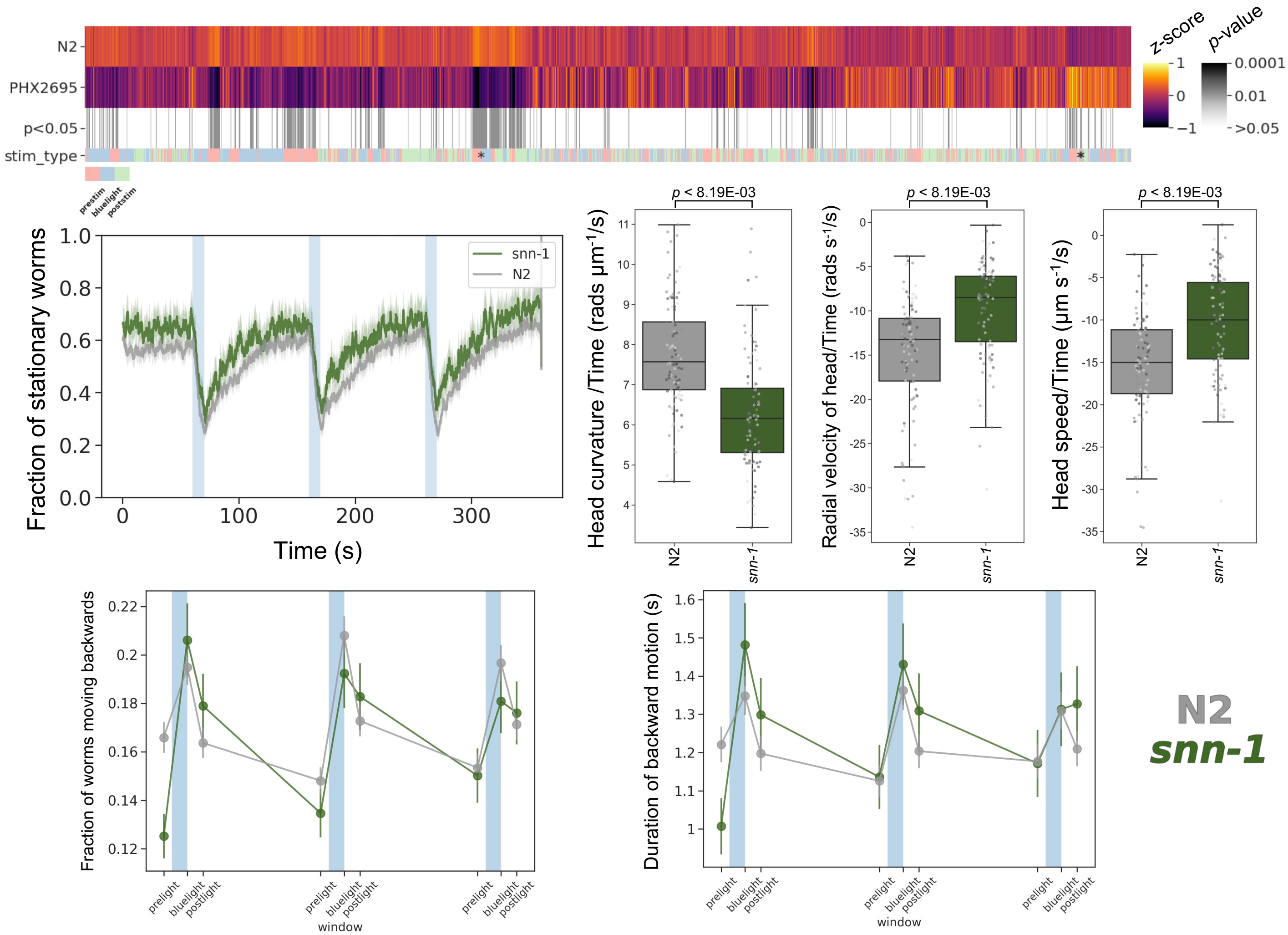

tmem-231(syb1575)

Strain name: PHX1575
Wild-type gene length: 2033bp
Mutation: 1253bp deletion at position 354 (covering exon 1 to beginning of exon 7)

C. elegans Description

Function: Involved in non-motile cilium assembly

Expression: Amphid neurons; ciliated neurons; inner labial neurons; outer labial neurons and phasmid neurons

Previously reported phenotypes: No previous phenotype reported

Human Orthologs:

TMEM231

Associated disease(s):
Joubert syndrome 20, 614970 (AR); Meckel syndrome 11, 615397 (AR)

Results

811/8289 significant features vs N2 ($p < 0.05$, block permutation t-test, 10000 permutations)

Key phenotype(s): Slightly less active during peristimulus recording; increased head curvature and angular velocity of tail (during peristimulus only); increased speed upon stimulation with blue light; sustained backwards escape response to blue light

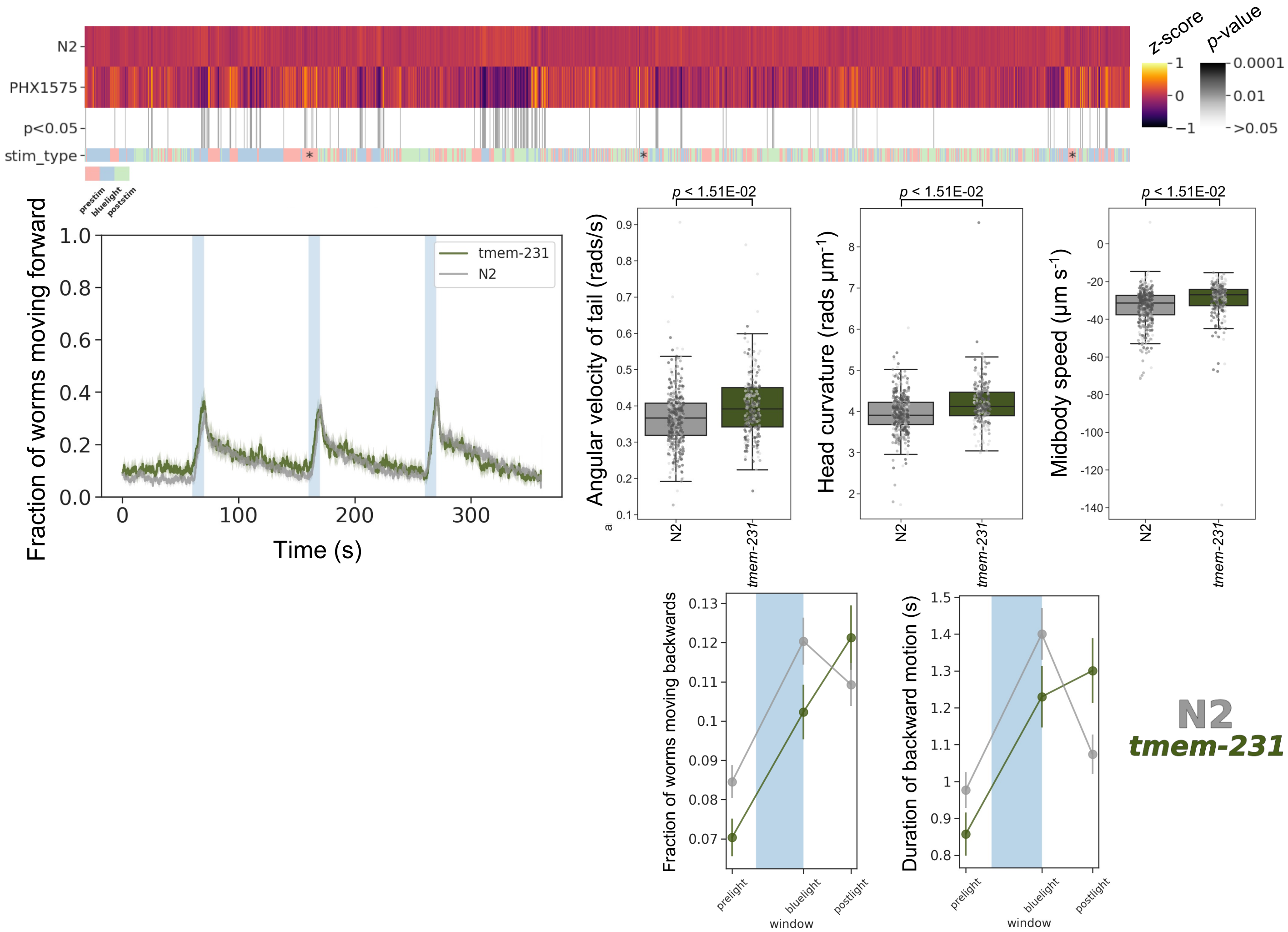

tub-1(syb1563)

Strain name: PHX1563
Wild-type gene length: 1964bp
Mutation: 1319bp deletion at position 3 (exon 1 to beginning of exon 4)

C. elegans Description

Function: Encodes TUBBY homolog and interacts with a 3-ketoacyl-coA thiolase, affecting lipid accumulation; involved in localisation of G-protein-coupled receptors in nonmotile cilia

Expression: Cytoplasm of ciliated cells

Previously reported phenotypes: Extended lifespan; increased fat content[24-25]

Human Orthologs:

TUB, TULP1

Associated disease(s):
Retinal dystrophy and obesity, 616188 (AR); Leber congenital amaurosis 15, 613843 (AR); Retinitis pigmentosa 14, 600132 (AR)

Results

4567/8289 significant features vs N2 ($p < 0.05$, block permutation t-test, 10000 permutations)

Key phenotype(s): Shorter and fatter; decreased curvature; deeper body bends (increased time derivative of curvature); hyperactive prior to simulation; decreased speed; attenuated, but sustained, sustained forward escape response; little difference in backwards motion/reversals

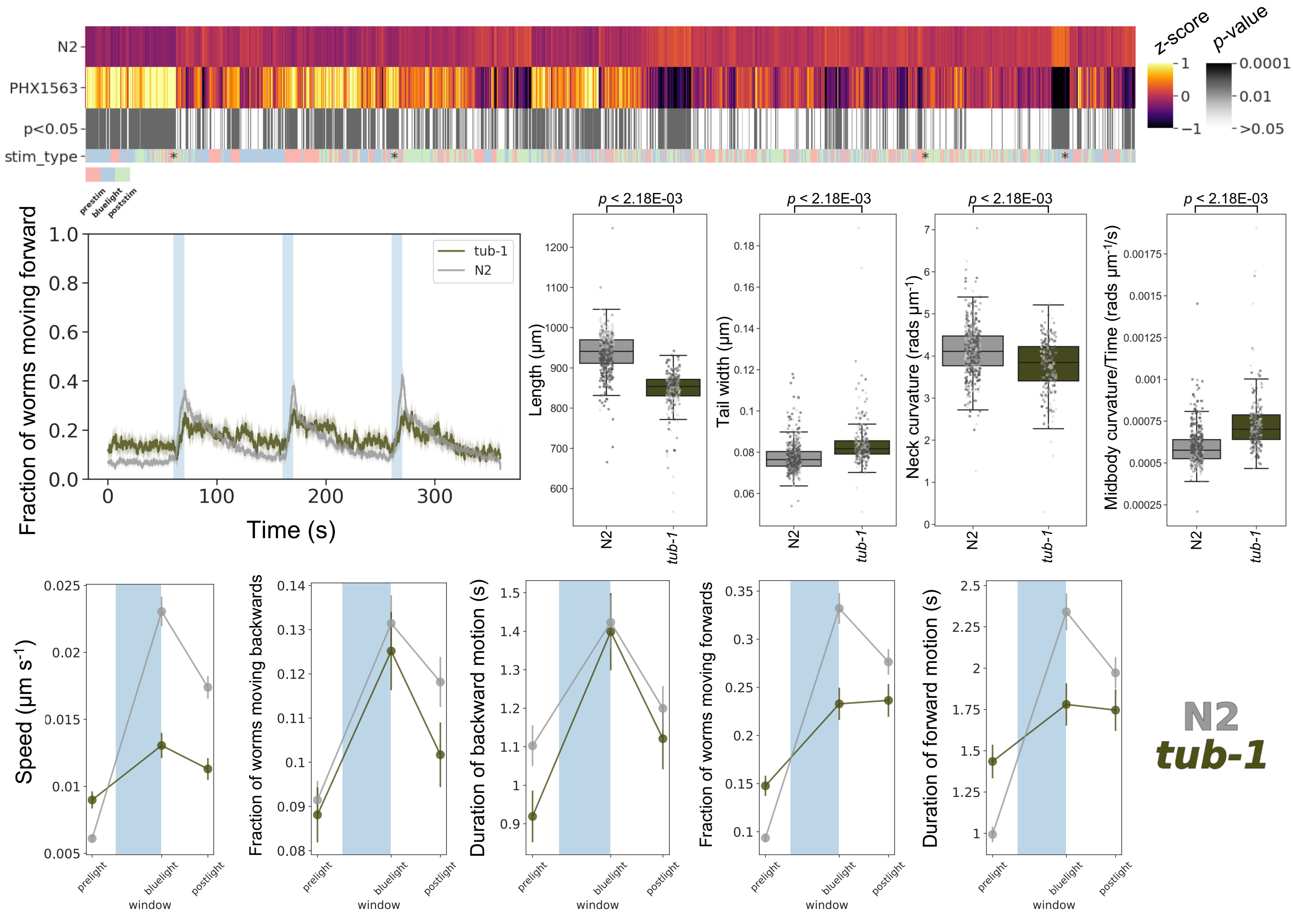

unc-25(syb1651)

Strain name: PHX1651
Wild-type gene length: 1706bp
Mutation: 1304bp deletion at position 638 (exons 2-4)

C. elegans Description

Function: Encodes glutamic acid decarboxylase (inolved in GABA biosynthesis)

Expression: EF neuron and neurons

Previously reported phenotypes: GABA deficient; shrinker; defective defecation; irregular head thrashes; sensitive to PTZ, fluoxetine and aldicarb[26-32]

Human Orthologs:

GAD1

Associated diseases(s):
Cerebral palsy, spastic quadriplegic, 1, 603513 (AR)

Results

1956/8289 significant features vs N2 ($p < 0.05$, block permutation t-test, 10000 permutations)

Key phenotype(s): Developmentally delayed (2 days); shorter; deeper sinusoidal bends (increased time derivative of curvature); less active and slower; delayed, but sustained forward escape response to blue light exposure

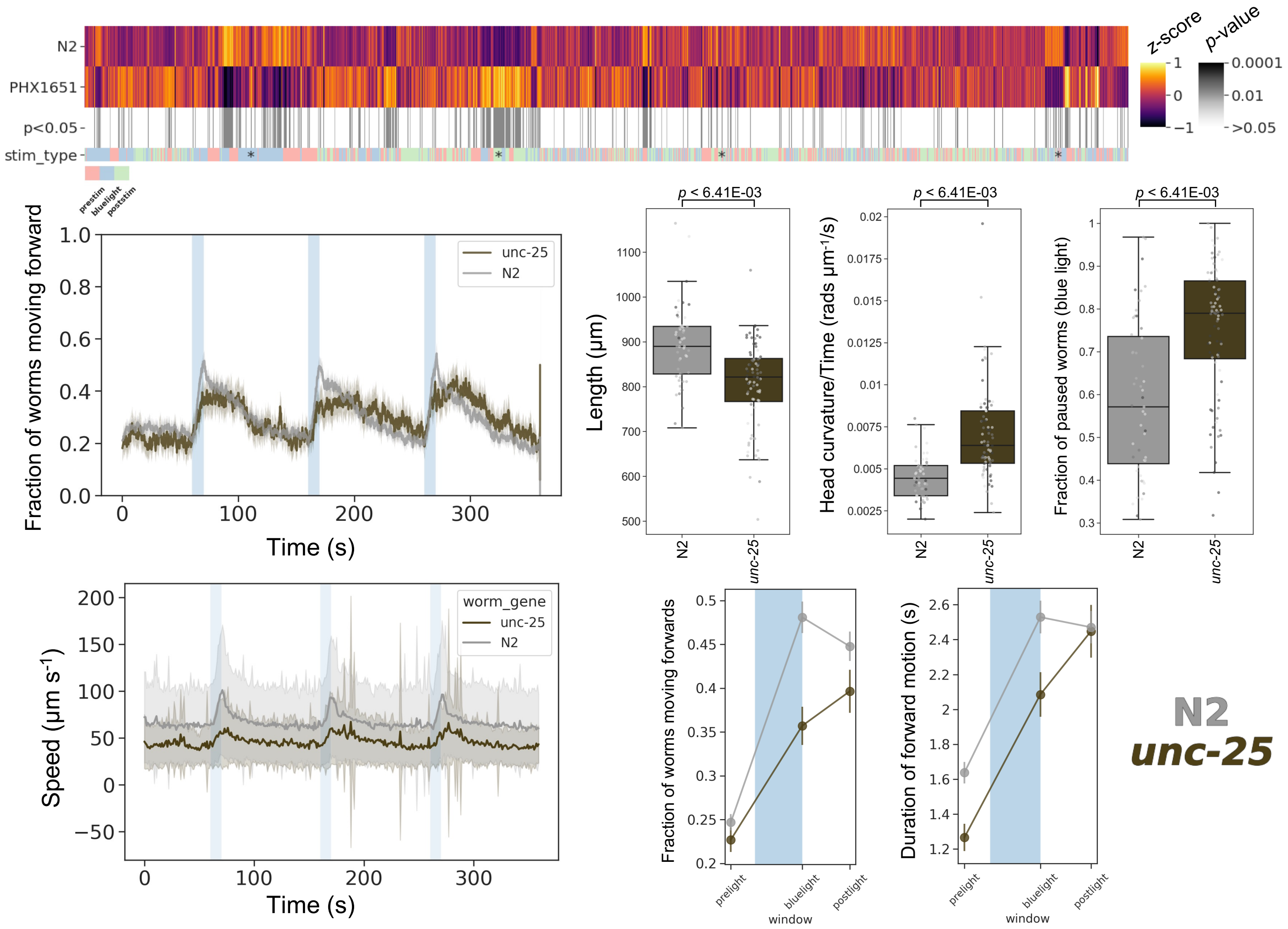

unc-43(syb2654)

Strain name: PHX2654
Wild-type gene length: 1823bp
Mutation: 1246bp deletion at position 157 (exon 1 to beginning of exon 3)

C. elegans Description

Function: Encodes CamKII-kinase; UNC-43 also interacts with the EGL-2 (ether-a-go-go potassium channel) to regulate it's activity

Expression: Neurons; oocytes; gonadal sheath cells; copulatory spicule

Previously reported phenotypes: Poor backing; slow moving; shrinker; defective egg laying; body wall muscle variant; sensitive to PTZ and aldicarb[30,33-36]

Human Orthologs:

CAMK2A; CAMK2B

Associated disease(s):
Intellectual disability, 53, 617798 (AD); Intellectual disability, 54, 617799 (AD)

Results

6858/8289 significant features vs N2 ($p < 0.05$, block permutation t-test, 10000 permutations)

Key phenotype(s): Small; increased curvature and head foraging while moving (time derivative of angular velocity); hyperactive; increased speed; increased and sustained escape response to blue light stimulation

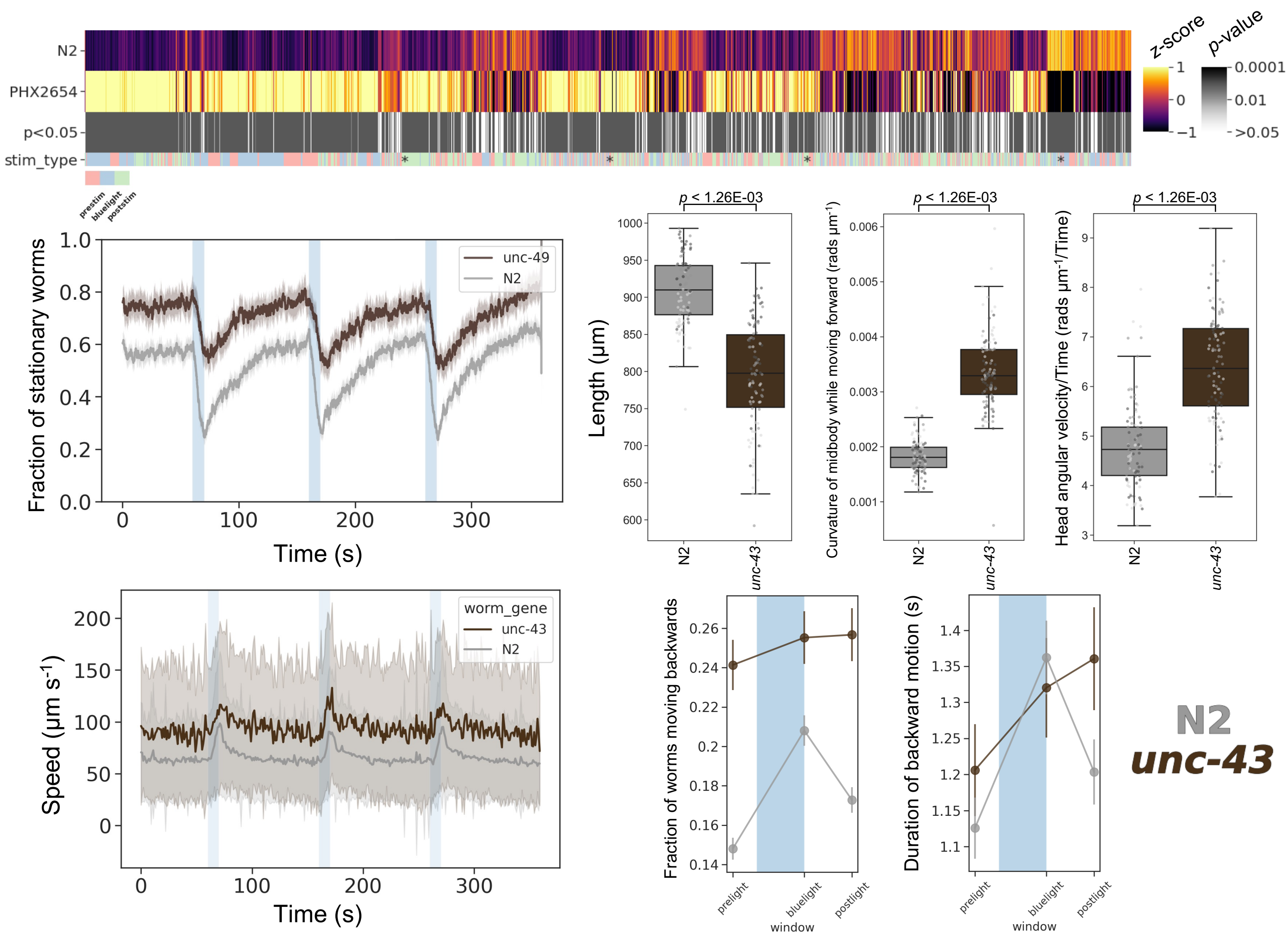

unc-49(syb2856)

Strain name: PHX2856
Wild-type gene length: 8713bp
Mutation: 7765bp deletion at position 567 (9 exons)

C. elegans Description

Function: Encodes subunits of heteromeric GABA receptor; UNC-49 is also required for postsynaptic GABA responsiveness

Expression: Expressed in anal sphincter muscle; body wall musculature; dorsal nerve cord; nerve ring and ventral nerve cord

Previously reported phenotypes: Decreased backing and thrashing; asymmetric body bends; small; shrinker; hypersensitive to fluoxetine and PTZ; resistant to muscimol[12,17-18,28,30-32,37-38]

Human Orthologs:

GABRB1; GABRB2; GABRB3

Associated disease(s):
Epileptic encephalopathy, early infantile, 43 and 45; Epileptic encephalopathy, infantile or early childhood, 2; Childhood absence epilepsy; Early onset epileptic encephalopathy

Results

4799/8289 significant features vs N2 ($p < 0.05$, block permutation t-test, 10000 permutations)

Key phenotype(s): Less active; smaller; decreased angular velocity of neck; increased curvature of midbody; decreased speed; sustained forward escape response, but attenuated backward escape response to blue light

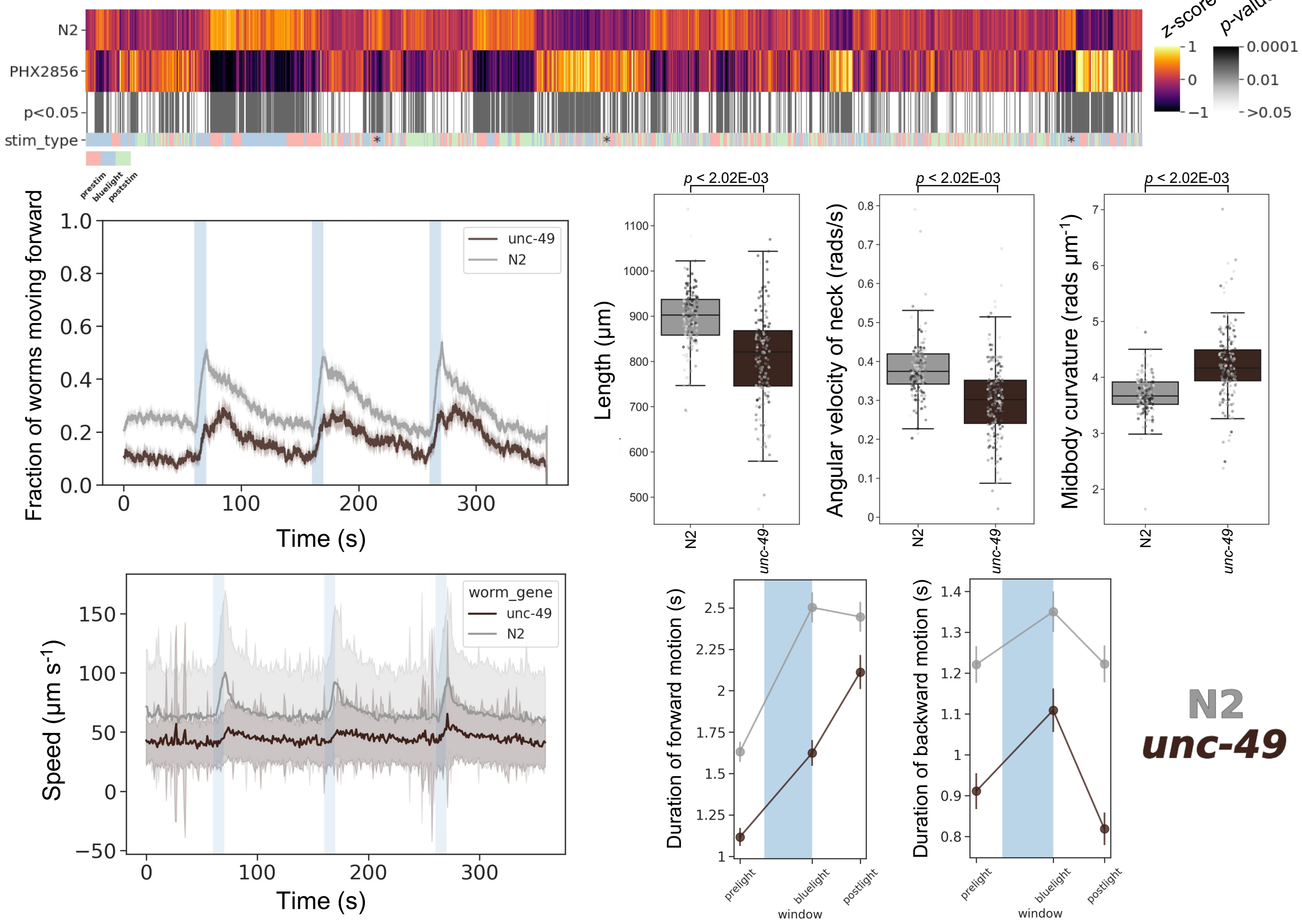

unc-77(syb1688)

Strain name: PHX1688
Wild-type gene length: 2046bp
Mutation: 1407bp deletion at position 8 (first 4 exons of NM171353.4)

C. elegans Description

Function: Cation-leak channel; interacts with *unc-80*, *unc-79* and *nca-2*

Expression: Cholinergic neurons; nerve ring; pharyngeal neurons and tail ganglion

Previously reported phenotypes: Decreased backing and increased sinusoidal movement[15]

Human Orthologs:

NALCN

Associated disease(s):
Congenital contractures of the limbs and face, hypotonia, and developmental delay, 616266 (AD); Hypotonia, infantile, with psychomotor retardation and characteristic facies 1, 615419 (AR)

Results

1011/8289 significant features vs N2 (p < 0.05, block permutation t-test, 10000 permutations)

Key phenotype(s): Decreased curvature of head/neck; stronger increase in speed in response to blue light; defective backing response, characterised by: decreased rate/duration of reversals, low frequency back bends (time derivative of curvature) and increase in time derivative of angular velocity related features across the body

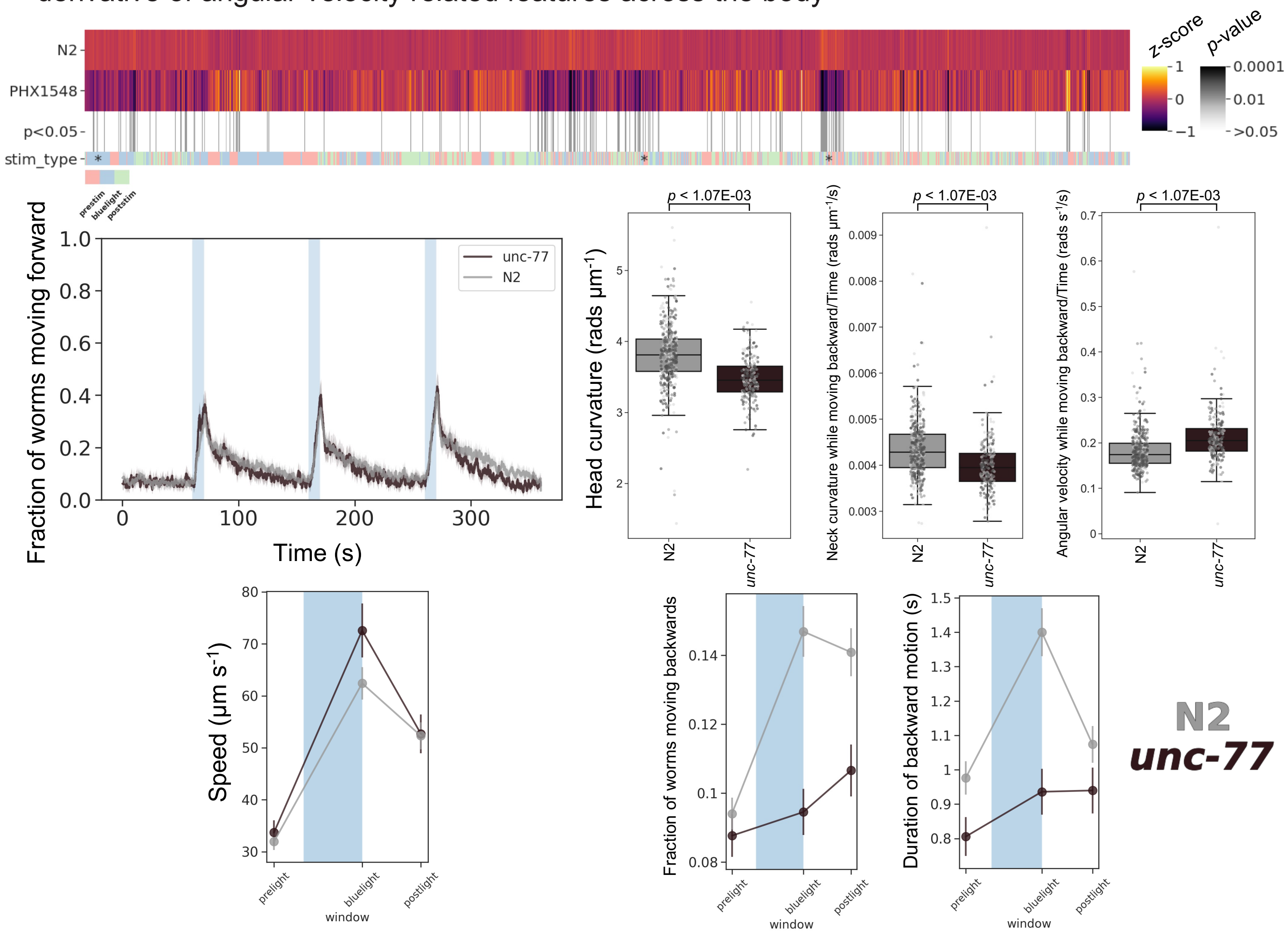

unc-80(syb1531)

Strain name: PHX1531
Wild-type gene length: 1806bp
Mutation: 1367bp deletion at position 60 (second half of exon 1 to middle of exon 4)

C. elegans Description

Function: Cation-leak channel; interacts with *unc-77*, *unc-79* and *nca-2*

Expression: Cholinergic neurons; excretory canal; hermaphrodite distal tip cell; non-striated muscle and somatic nervous system

Previously reported phenotypes: Sinusoidal, coiling and locomotion phenotypes; fainting in liquid; sensitivity to halothane, isoflurane and ethanol[15,39-40]

Human Orthologs:

UNC80

Associated disease(s):
Hypotonia, infantile, with psychomotor retardation and characteristic facies 2, 616801 (AR)

Results

4868/8289 significant features vs N2 ($p < 0.05$, block permutation t-test, 10000 permutations)

Key phenotype(s): Shorter; decreased curvature and speed; less active; attenuated blue light response (e.g., lack of backward escape response); and fainting (increased pausing) following cessation of blue light stimulation

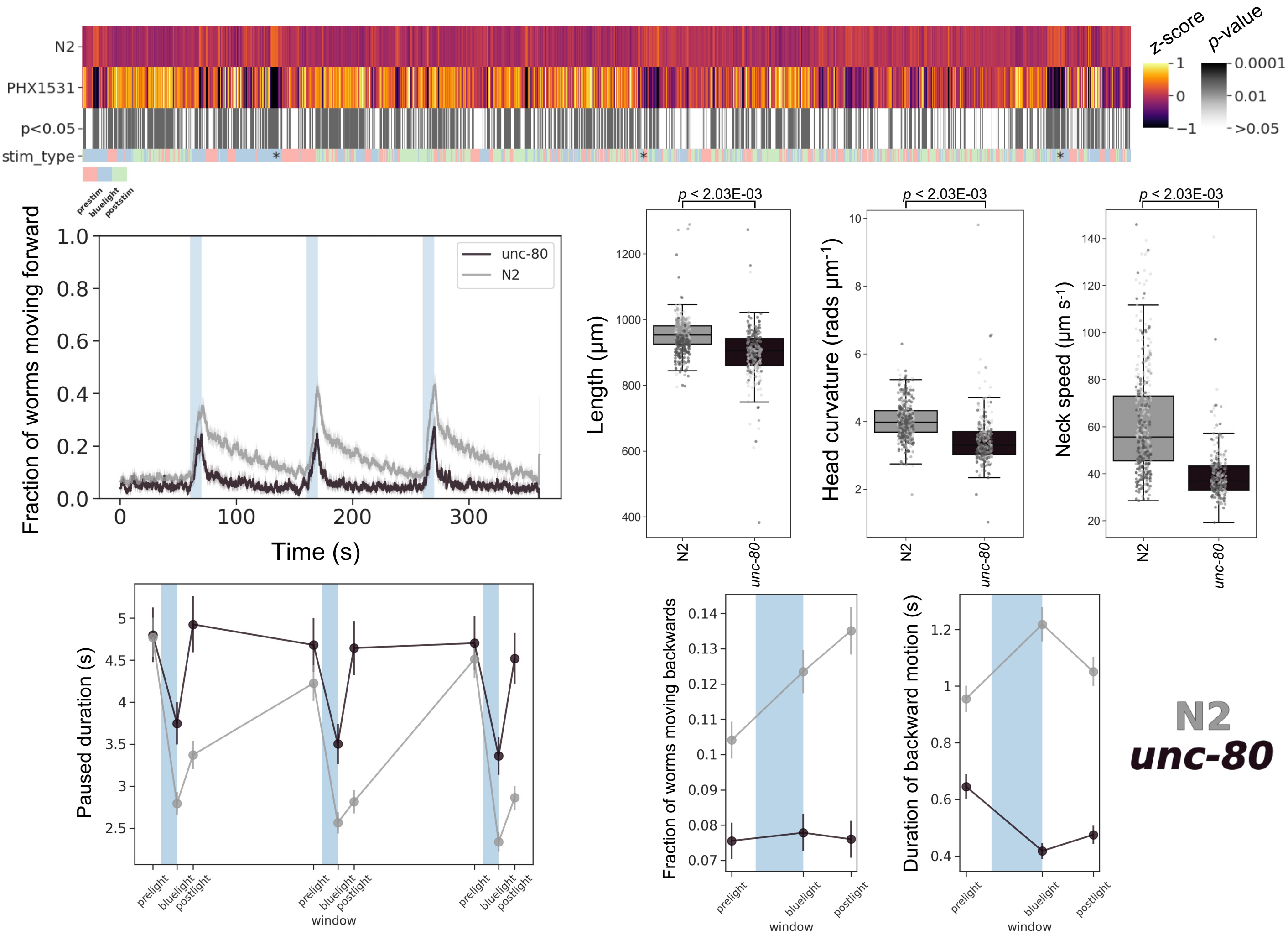

References

[1]Vukojevic V., Gschwind L., Vogler C., *et al.* (2012), A role for α-adducin (ADD-1) in nematode and human memory. The EMBO Journal, 31:1453-1466. doi:10.1038/emboj.2012.14

[2]Flavell S.W., Pokala N., Macosko E.Z., *et al.* (2013) Serotonin and the Neuropeptide PDF Initiate and Extend Opposing Behavioral States in *C. elegans. Cell*. 154(5):1023-1035. doi:10.1016/j.cell.2013.08.001

[3]Kuramochi M. and Doi M. (2019) An Excitatory/Inhibitory Switch From Asymmetric Sensory Neurons Defines Postsynaptic Tuning for a Rapid Response to NaCl in *Caenorhabditis elegans*. *Front Mol Neurosci*. 11:484. doi:10.3389/fnmol.2018.00484

[4]Lee B.H, Liu J., Wong D., *et al.* (2011) Hyperactive neuroendocrine secretion causes size, feeding, and metabolic defects of *C. elegans* Bardet-Biedl syndrome mutants. PLoS Biol. 9(12):e1001219. doi:10.1371/journal.pbio.1001219

[5]Page A.P. (2018) The sensory amphidial structures of *Caenorhabditis elegans* are involved in macrocyclic lactone uptake and anthelmintic resistance. *Int J Parasitol*. 48(13):1035-1042. doi:10.1016/j.ijpara.2018.06.003

[6]Omura D.T., Clark D.A., Samuel A.D. and Horvitz H.R. (2012). Dopamine signaling is essential for precise rates of locomotion by *C. elegans*. *PLoS One*. 7:e38649. doi:10.1371/journal.pone.0038649

[7]Sawin E.R., Ranganathan R. and Horvitz R.H. (2000) *C. elegans* Locomotory Rate Is Modulated by the Environment through a Dopaminergic Pathway and by Experience through a Serotonergic Pathway. *Neuron*. 26(3):619-631. doi:10.1016/S0896-6273(00)81199-X

[8]Beron C., Vidal-Gadea A.G., Cohn J., *et al.* (2015) The burrowing behavior of the nematode Caenorhabditis elegans: a new assay for the study of neuromuscular disorders. *Genes Brain Behav*. 14, 357-68. doi:10.1111/gbb.12217

[9]Gieseler K., Bessou C., and Segalat L.S. (1999). Dystrobrevin- and dystrophin-like mutants display similar phenotypes in the nematode *Caenorhabditis elegans*. *Neurogenetics*. 2:87-90. doi:10.1007/s100480050057

[10]Hills T., Brockie P.J. and Maricq A.V. (2004). Dopamine and glutamate control area-restricted search behavior in *Caenorhabditis elegans*. *J Neurosci*, 24:1217-25. doi:10.1523/JNEUROSCI.1569-03.2004

[11]Kullyev A., Dempsey C.M., Miller S., *et al.* (2010). A genetic survey of fluoxetine action on synaptic transmission in *Caenorhabditis elegans*. *Genetics*, 186:929-41. doi:10.1534/genetics.110.118877

[12]Zheng Y., Brockie P.J, Mellem J.E., *et al.* (1999) Neuronal Control of Locomotion in *C. elegans* Is Modified by a Dominant Mutation in the GLR-1 Ionotropic Glutamate Receptor. *Neuron*. 24(2):346-361. doi:10.1016/S0896-6273(00)80849-1

[13]Sieburth D., Ch'ng Q., Dybbs M., *et al.* (2005). Systematic analysis of genes required for synapse structure and function. *Nature*, 436:510-7. doi:10.1038/nature03809

[14]Avery L. (1993) The genetics of feeding in *Caenorhabditis elegans*. *Genetics*. 133(4):897-917. doi:10.1093/genetics/133.4.897.

[15]Yemini E., Jucikas T., Grundy L.J., Brown A.E. and Schafer W.R. (2013) A database of *Caenorhabditis elegans* behavioral phenotypes. *Nat Methods*. 10(9):877-9 doi:10.1038/nmeth.2560

[16]You Y.J., Kim J., Cobb M. and Avery L. (2006) Starvation activates MAP kinase through the muscarinic acetylcholine pathway in *Caenorhabditis elegans* pharynx. *Cell Metab*. 3(4):237-245. doi:10.1016/j.cmet.2006.02.012

[17]Bellemer A., Hirata T., Romero M.F., and Koelle M.R. (2011). Two types of chloride transporters are required for GABA(A) receptor-mediated inhibition in *C. elegans*. *EMBO J*. 30:1852-63. doi:10.1038/emboj.2011.83

[18]Tanis J.E., Bellemer A., Moresco J.J., Forbush B. and Koelle M.R. (2009). The potassium chloride cotransporter KCC-2 coordinates development of inhibitory neurotransmission and synapse structure in *Caenorhabditis elegans*. *J Neurosci*. 29:9943-54. doi:10.1523/JNEUROSCI.1989-09.2009

[19]Xiao H., Hapiak V.M., Smith K.A., *et al.* (2006). SER-1, a *Caenorhabditis elegans* 5-HT2-like receptor, and a multi-PDZ domain containing protein (MPZ-1) interact in vulval muscle to facilitate serotonin-stimulated egg-laying. *Dev Biol*. 298:379-91. doi:10.1016/j.ydbio.2006.06.044

[20]Bess A.S., Crocker T.L., Ryde I.T., and Meyer J.N. (2012). Mitochondrial dynamics and autophagy aid in removal of persistent mitochondrial DNA damage in *Caenorhabditis elegans*. *Nucleic Acids Res*. 40:7916-31. doi:10.1093/nar/gks532

[21]Bornhorst J., Chakraborty S., Meyer S., *et al*. (2014). The effects of *pdr1*, *djr1.1* and *pink1* loss in manganese-induced toxicity and the role of alpha-synuclein in *C. elegans*. *Metallomics*. 6:476-90. doi:10.1039/c3mt00325f

[22]Sämann J., Hegermann J., von Gromoff E., (2009) *Caenorhabditits elegans* LRK-1 and PINK-1 act antagonistically in stress response and neurite outgrowth. *J Biol Chem*. 284(24):16482-16491. doi:10.1074/jbc.M808255200.

[23]Stavoe A.K., Nelson J.C., Martinez-Velazquez L.A., *et al*. (2012). Synaptic vesicle clustering requires a distinct MIG-10/Lamellipodin isoform and ABI-1 downstream from Netrin. *Genes Dev*. 26:2206-21. doi:10.1101/gad.193409.112

[24]Lemieux G.A., Liu J., Mayer N., *et al*. (2011). A whole-organism screen identifies new regulators of fat storage. *Nat Chem Biol*. 7:206-13. doi:10.1038/nchembio.534

[25]Mak H.Y., Nelson L.S., Basson M., Johnson C.D. and Ruvkun G. (2006). Polygenic control of *Caenorhabditis elegans* fat storage. *Nat Genet*. 38:363-8. doi:10.1038/ng1739

[26]Jin Y., Jorgensen E., Hartwieg E. and Horvitz H.R. (1999) The *Caenorhabditis elegans* gene *unc-25* encodes glutamic acid decarboxylase and is required for synaptic transmission but not synaptic development. *J Neurosci*. 19(2):539-48. doi:10.1523/JNEUROSCI.19-02-00539.1999.

[27]Kullyev A., Dempsey C.M., Miller S., *et al*. (2010). A genetic survey of fluoxetine action on synaptic transmission in *Caenorhabditis elegans*. *Genetics*. 186:929-41. doi:10.1534/genetics.110.118877

[28]Locke C.J., Kautu B.B., Berry K.P., *et al*. (2009). Pharmacogenetic analysis reveals a post-developmental role for Rac GTPases in *Caenorhabditis elegans* GABAergic neurotransmission. *Genetics*. 183:1357-72. doi:10.1534/genetics.109.106880

[29]Loria P.M., Duke A., Rand J.B. and Hobert O. (2003). Two neuronal, nuclear-localized RNA binding proteins involved in synaptic transmission. *Curr Biol*. 13:1317-23. doi:10.1016/S0960-9822(03)00532-3

[30]Wong S.Q, Jones A., Dodd S., *et al*. (2018). A *Caenorhabditis elegans* assay of seizure-like activity optimised for identifying antiepileptic drugs and their mechanisms of action. *J Neurosci Methods*. 309:132-142. doi:10.1016/j.jneumeth.2018.09.004

[31]Shingai R., Furudate M., Hoshi K., and Iwasaki Y. (2013). Evaluation of Head Movement Periodicity and Irregularity during Locomotion of *Caenorhabditis elegans*. *Front Behav Neurosci*. 7:20. doi:10.3389/fnbeh.2013.00020

[32]Singh K., Ju J.Y., Walsh M.B., DiIorio M.A. and Hart A.C. (2014). Deep conservation of genes required for both *Drosphila melanogaster* and *Caenorhabditis elegans* sleep includes a role for dopaminergic signaling. *Sleep*. 37:1439-51. doi:10.5665/sleep.3990

[33]Harrington S., Knox J.J., Burns A.R. *et al*. (2022) Egg-laying and locomotory screens with *C. elegans* yield a nematode-selective small molecule stimulator of neurotransmitter release. *Commun Biol*. 5:865. doi:10.1038/s42003-022-03819-6

[34]LeBoeuf B., Gruninger T.R. and Garcia L.R. (2007). Food deprivation attenuates seizures through CaMKII and EAG K+ channels. *PLoS Genet*. 3:1622-32. doi:10.1371/journal.pgen.0030156

[35]Reiner D.J., Weinshenker D.Z. and Thomas J.H. (1995). Analysis of dominant mutations affecting muscle excitation in *Caenorhabditis elegans*. *Genetics*. 141:961-76. doi:10.1093/genetics/141.3.961

[36]Vashlishan A.B., Madison J.M., Dybbs M., *et al*. (2008). An RNAi screen identifies genes that regulate GABA synapses. *Neuron*. 58:346-61. doi:10.1016/j.neuron.2008.02.019

[37]Bamber B.A., Beg A.A., Twyman R.E. and Jorgensen E.M. (1999). The *Caenorhabditis elegans unc-49* locus encodes multiple subunits of a heteromultimeric GABA receptor. *J Neurosci*. 19:5348-59. doi:10.1523/JNEUROSCI.19-13-05348.1999

[38]Liewald J.F., Brauner M., Stephens G.J., *et al*. (2008). Optogenetic analysis of synaptic function. *Nat Methods*. 5:895-902. doi:10.1038/nmeth.1252

[39]Morgan P.G. and Sedensky M.M. (1994). Mutations conferring new patterns of sensitivity to volatile anesthetics in *Caenorhabditis elegans*. *Anesthesiology*. 81:888-98. doi:10.1097/00000542-199410000-00016

[40]Sedensky M.M. and Meneely P.M. (1987). Genetic analysis of halothane sensitivity in *Caenorhabditis elegans*. *Science*. 236:952-4. doi:10.1126/science.3576211
